# Water‐suppression cycling 3‐T cardiac ^1^H‐MRS detects altered creatine and choline in patients with aortic or mitral stenosis

**DOI:** 10.1002/nbm.4513

**Published:** 2021-04-07

**Authors:** Belinda Ding, Mark Peterzan, Ferenc E. Mózes, Oliver J. Rider, Ladislav Valkovič, Christopher T. Rodgers

**Affiliations:** ^1^ Wolfson Brain Imaging Centre University of Cambridge Cambridge UK; ^2^ Oxford Centre for Clinical Magnetic Resonance Research (OCMR) University of Oxford Oxford UK; ^3^ Department of Imaging Methods, Institute of Measurement Science Slovak Academy of Sciences Bratislava Slovakia

**Keywords:** ^1^H‐MRS, 3 T, cardiac, heart, human, PRESS, STEAM, water‐suppression cycling

## Abstract

Cardiac proton spectroscopy (^1^H‐MRS) is widely used to quantify lipids. Other metabolites (e.g. creatine and choline) are clinically relevant but more challenging to quantify because of their low concentrations (approximately 10 mmol/L) and because of cardiac motion. To quantify cardiac creatine and choline, we added water‐suppression cycling (WSC) to two single‐voxel spectroscopy sequences (STEAM and PRESS). WSC introduces controlled residual water signals that alternate between positive and negative phases from transient to transient, enabling robust phase and frequency correction. Moreover, a particular weighted sum of transients eliminates residual water signals without baseline distortion. We compared WSC and the vendor's standard ‘WET’ water suppression in phantoms. Next, we tested repeatability in 10 volunteers (seven males, three females; age 29.3 ± 4.0 years; body mass index [BMI] 23.7 ± 4.1 kg/m^2^). Fat fraction, creatine concentration and choline concentration when quantified by STEAM‐WET were 0.30% ± 0.11%, 29.6 ± 7.0 μmol/g and 7.9 ± 6.7 μmol/g, respectively; and when quantified by PRESS‐WSC they were 0.30% ± 0.15%, 31.5 ± 3.1 μmol/g and 8.3 ± 4.4 μmol/g, respectively. Compared with STEAM‐WET, PRESS‐WSC gave spectra whose fitting quality expressed by Cramér‐Rao lower bounds improved by 26% for creatine and 32% for choline. Repeatability of metabolite concentration measurements improved by 72% for creatine and 40% for choline. We also compared STEAM‐WET and PRESS‐WSC in 13 patients with severe symptomatic aortic or mitral stenosis indicated for valve replacement surgery (10 males, three females; age 75.9 ± 6.3 years; BMI 27.4 ± 4.3 kg/m^2^). Spectra were of analysable quality in eight patients for STEAM‐WET, and in nine for PRESS‐WSC. We observed comparable lipid concentrations with those in healthy volunteers, significantly reduced creatine concentrations, and a trend towards decreased choline concentrations. We conclude that PRESS‐WSC offers improved performance and reproducibility for the quantification of cardiac lipids, creatine and choline concentrations in healthy volunteers at 3 T. It also offers improved performance compared with STEAM‐WET for detecting altered creatine and choline concentrations in patients with valve disease.

## Graphical abstract

A water‐suppression cycling (WSC) module was added to STEAM and PRESS single‐voxel spectroscopy pulse sequences. We report an improvement in the accuracy and repeatability in the quantification of low concentration metabolites like creatine and choline in the myocardium using WSC compared with the vendor's standard WET water suppression. This allowed us to detect a significant decrease in creatine levels in patients with symptomatic aortic stenosis in less than half the scan time compared with the vendor's standard WET water suppression.

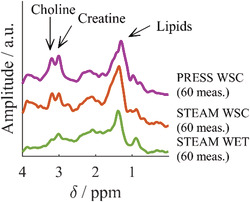

**Abbreviations used**
ADP: adenosine diphosphate;
ATP: adenosine triphosphate
Cho: choline
CK: creatine kinase
Cr: creatine
CRLB: Cramér‐Rao lower bounds
HLSVD: Hankel–Lanczos singular value decomposition
MAD: median absolute deviations
OXSA: Oxford Spectroscopy Analysis (open‐source MATLAB toolbox)
PCr: phosphocreatine
P_i_: inorganic phosphate
PRESS: Point RESolved Spectroscopy
STEAM: STimulated Echo Acquisition Mode
WET: water suppression enhanced through T_1_ effects
WSC: water‐suppression cycling

## 1 INTRODUCTION

Magnetic resonance spectroscopy (^1^H‐MRS) is an established method to assess metabolite concentrations in the human heart. In diseases such as obesity and type 2 diabetes, the heart's uptake and oxidation of fatty acids are not balanced, resulting in an accumulation of triglycerides.^1^ The accumulation of triglycerides leads to metabolic derangement, which has been proposed as a contributory and/or perpetuating factor in nonischaemic cardiomyopathies.^1^, ^2^ Therefore, most published cardiac ^1^H‐MRS studies have focused on quantifying lipid concentrations.^3^, ^4^, ^5^ However, with sufficient data quality, ^1^H‐MRS can also assess lower concentration metabolites such as creatine (Cr) and choline* (Cho) that are of high clinical interest.^6^, ^7^, ^8^

Cr is an important component in the creatine kinase (CK) enzyme system.^9^ CK catalyses the transfer of a high‐energy phosphate group from adenosine triphosphate (ATP) onto Cr to form phosphocreatine (PCr) and adenosine diphosphate (ADP) in the mitochondria:

(1) ATP+freeCr⇌ADP+PCr.

In the myofibrils, the reverse reaction provides a very rapid supply of ATP to meet demand during bursts of effort such as sustaining contraction throughout systole.^10^, ^11^ These ATP‐delivery processes become deranged in heart failure, according to the energy‐starvation hypothesis.^12^ In failing human and animal hearts, free Cr and PCr concentrations drop substantially soon after disease onset^13^ and before failure of ATP homeostasis.^9^, ^14^ Note that, in vivo, the free Cr and PCr ^1^H‐MRS peaks are overlapping. It is therefore common to report ‘total creatine’ (tCr), defined as the sum of free Cr and PCr. Cr concentrations obtained via ^1^H‐MRS can be used to calculate the heart's energy reserve (∣ΔG_ATP_∣**)**,^15^, ^16^ which in turn can predict isovolumic contractile reserve^17^ and peak cardiac work.^18^, ^19^ Hence, a decrease in ∣ΔG_ATP_∣ limits the heart's maximum mechanical work and, eventually, a reduced ∣ΔG_ATP_ ∣ could lead to heart failure. On top of that, ∣ΔG_ATP_∣ is a potential biomarker that could be used to evaluate novel ‘energy‐sparing’ treatments for heart failure, for example, drugs that modulate fatty acid oxidation, such as etomoxir^20^ and perhexiline.^21^

Cholines are a class of quaternary ammonium salts that serve several physiological functions such as maintaining structural integrity in cell membranes and facilitating cell membrane signalling.^22^ The Cho derivative acetylcholine is an important neurotransmitter in the autonomic nervous system, which regulates heart rate and blood pressure,^23^ and in the brain, where defective cholinergic signalling is implicated in Alzheimer's dementia.^24^ Cholines have also been identified as a potential biomarker for identifying active tumours, for example, in the brain,^25^ breast^26^ and liver.^27^ However, whether myocardial choline levels change in disease is still poorly understood in both animal models and humans.

To date, most in vivo cardiac ^1^H‐MRS studies have used single‐voxel spectroscopy (SVS) techniques such as STimulated Echo Acquisition Mode (STEAM) and Point RESolved Spectroscopy (PRESS) together with a pulse sequence module that suppresses water signals.^28^ Patient motion, including respiration and cardiac motion, mean that there can be phase and frequency shifts between each transient recorded by the scanner. This can cause incoherent averaging during postprocessing, and hence reduce signal‐to‐noise ratio (SNR). A common approach for both cardiac and brain spectroscopy involves phasing and frequency shifting individual transients using the largest peak (residual water or a metabolite).^3^, ^28^, ^29^, ^30^, ^31^, ^32^ To ensure that there is a sufficiently large reference peak for frequency and phase correction, many studies only partially suppress water (so‐called ‘weak water suppression’).^28^, ^29^ However, with weak water suppression, the residual water peak can still cause problems during spectral fitting.^33^

In this work, we propose instead to use a water‐suppression cycling (WSC) method,^34^ originally developed for brain ^1^H‐MRS, to improve the quality of cardiac single‐voxel ^1^H‐MRS at 3 T. The WSC technique inserts a weak water suppression module and adds an inversion pulse in alternate transients. The weak water suppression property of this module provides a high SNR residual water peak for frequency and phase correction, while inverting the residual water signal every other scan allows for practically complete elimination of the residual water peak during postprocessing (as we show below), which improves spectral fitting (e.g. by avoiding errors from baseline distortion). WSC is similar conceptually to the method known as ‘metabolite cycling’,^7^, ^35^ except that during metabolite cycling it is the metabolite peaks that are inverted, whereas WSC only inverts the residual water peak.

Here, we aimed to test the feasibility of the WSC approach to improve quantification of low‐concentration metabolites such as Cr and Cho (as well as lipids) in the heart at 3 T; and to investigate potential changes in cardiac Cr and Cho levels in patients with valvular stenosis.

This is a particularly exciting, but as of yet underinvestigated group of patients, in which 15% of patients undergoing surgical aortic valve replacement have reduced left ventricular ejection fraction (LVEF; <50%).^36^, ^37^, ^38^However, at present it is not possible to anticipate or explain why the myocardium in some patients, but not others, exhibits contractile decline in the face of sustained severe pressure overload. Nonetheless, otherwise unexplained contractile decline into the borderline (LVEF 50%–59%) range worsens prognosis^38^, ^39^, ^40^ and, in the absence of good understanding regarding the underlying mechanism, there is currently no specific medical therapy other than to refer patients for timely valve replacement once the valve disease has progressed into the severe range. The metabolic hypothesis of heart failure posits that reduced metabolic reserve is both a permissive and a causative factor at play, and while there are many animal models that support this,^41^, ^42^ the evidence in human pressure overload hypertrophy is sparser^13^, ^43^ because of the difficulty in obtaining human myocardium, and also because there are few established techniques for noninvasive myocardial metabolic phenotyping. Despite the known association between reduced PCr/ATP ratio and pathological left ventricular hypertrophy, there is mixed evidence regarding whether tCr itself is reduced in this setting,^43^, ^44^, ^45^, ^46^, ^47^ yet this would be a key variable to establish if one is to estimate the concentration of free ADP from the CK equilibrium expression and thereby come to an estimate of the free energy of ATP hydrolysis.

## 2 THEORY

### 2.1 Water‐suppression cycling

The water‐suppression cycling (WSC) module was first implemented by Ernst and Li for ^1^H‐MRS in the brain.^34^ This technique alternates between positive and negative residual water signal every other transient. The residual water signal can be used to correct for phase and frequency shifts in single shots and its alternating phase allows for the elimination of the water peak upon postprocessing.

**FIGURE 1:**
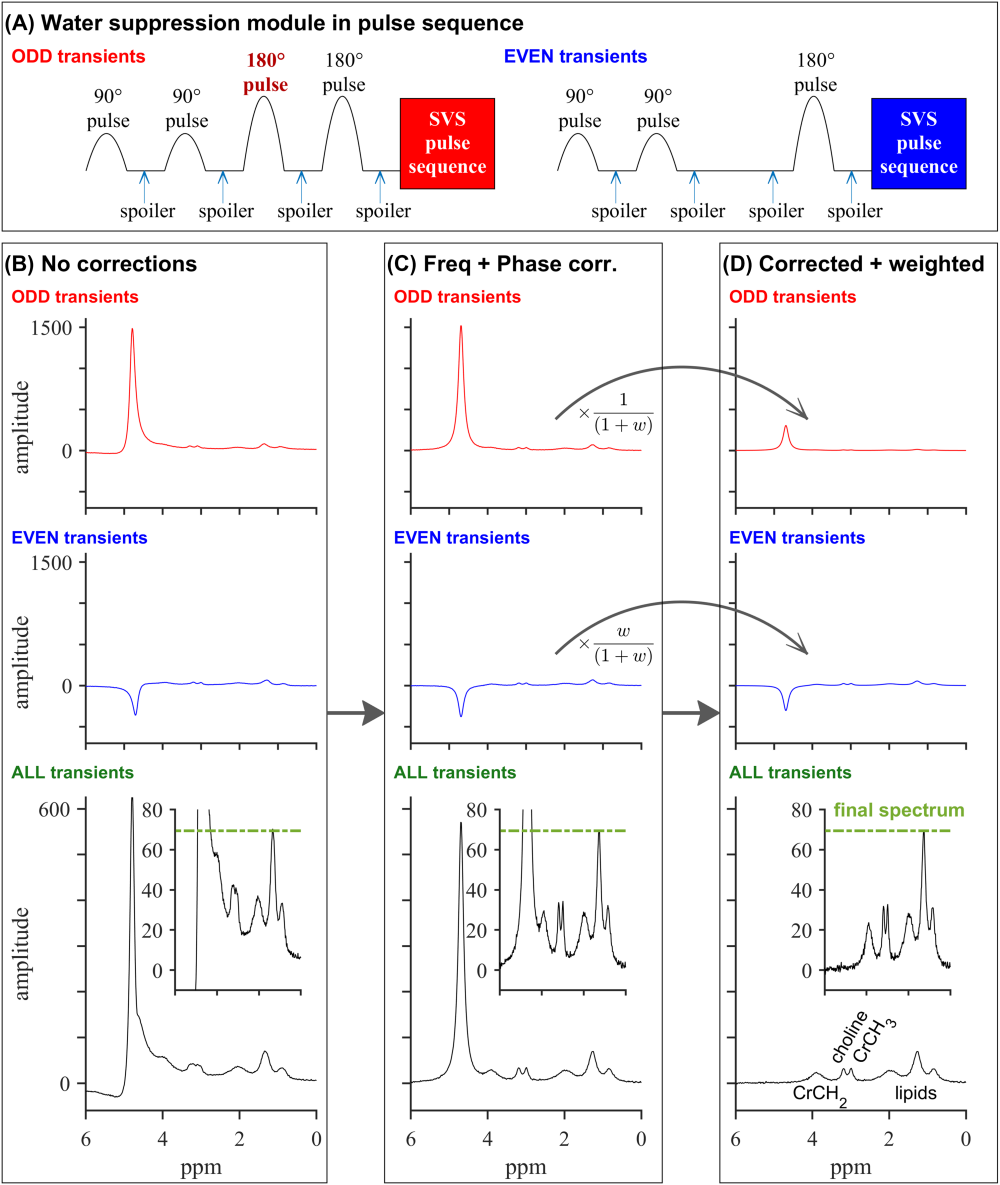
Explanation of the water‐suppression cycling (WSC) ^1^H‐MRS approach. A, Simplified pulse diagram showing the difference in water suppression between odd‐ and even‐numbered transients. B, Top: frequency domain plots of simulated example odd and even transients; bottom: the average of these; inset: zoom to show metabolite signal amplitudes. C, Equivalent plots after per‐transient frequency and phase correction based on the residual water peak. D, Equivalent plots with per‐transient frequency and phase correction and weighting according to Equation 2. The final WSC spectrum is shown at the bottom right. Note that a green dashed line is plotted at the same vertical position in all three columns to illustrate that the WSC processing preserves metabolite peak amplitudes

The implementation of the pulse sequence and the postprocessing of each transient is presented in Figure 1. WSC is an interleaved method. The WSC module comprises four chemical shift selective RF pulses spaced 50 ms apart for partial water suppression prior to a standard single‐voxel pulse sequence such as PRESS or STEAM. Each RF pulse is a Gaussian with a time‐bandwidth product of 0.896 centred on water, and the spoiler gradients between pulses suppress residual transverse magnetisation (M_xy_).^34^ The third pulse in the module, a 180° chemical shift selective inversion pulse, is activated in odd‐numbered acquisitions and disabled in even‐numbered acquisitions, yielding two different spectra, S_odd_ and S_even_. This alternation results in the residual water and metabolite peaks having the same phases in S_odd_ and opposite phases in S_even_.

Three corrections were applied before the odd‐ and even‐numbered acquisitions were averaged together. First, frequency correction was performed to correct for minor frequency differences between the water signals in each transient. Second, a zero‐order phase correction was performed based on the phase of the residual water peak to make it positive real in even transients and negative real in odd transients. Third, a weighting factor w, which compensates for T_1_ relaxation of the water signal and imperfections in the inversion pulses, was determined by least‐squares fitting, such that spectra summed according to Equation 2 have minimum residual water signal. Finally, the odd and even spectra were averaged using Equation 2, where the denominator ensures that metabolite amplitudes remain unchanged (see Figure S1 for simulation results):

(2) $$\begin{array}{c} S=\frac{S_{\mathrm{odd}}+w\times S_{\text{even}}}{1+w}\; \end{array}.$$

In many ways, the WSC approach is similar to the method of metabolite cycling.^35^ However, WSC has potential advantages, such as the residual water peak is eliminated before spectral fitting, and that because the metabolites are not inverted, WSC may be less sensitive to the T_1_ values of metabolites and the efficiency of the inversion pulse, as shown in Ernst and Li′s original study.^34^

## 3 METHODS

All data were collected using a whole‐body clinical 3 T Prisma MRI scanner (Siemens Healthineers, Erlangen, Germany) with an 18‐channel cardiac receive array coil (Siemens Healthineers) and a 32‐channel spine receive array coil (Siemens Healthineers).

### 3.1 ^1^H MRS sequence

We compared the vendor's standard ‘water suppression enhanced through T_1_ effects’ (WET)^48^ water‐suppression module against the WSC module described above. This gave four different protocols:

1. STEAM‐WET
2. PRESS ‐WET
3. STEAM‐WSC
4. PRESS‐WSC.

To minimise the loss of signal due to T_2_ decay, we also modified the gradient slew rates and amplitudes to achieve shorter echo times for all four sequences (26 ms for PRESS and 10 ms for STEAM). On top of that, to improve performance of the vendor's WET water suppression for in vivo scans, we followed the procedure of Rial et al.^3^ and used an additional breath‐held prescan to manually optimise the water‐suppression flip angles.

### 3.2 Data acquisition

#### 3.2.1 Phantom study

A phantom study was conducted to validate the ability to quantify Cr concentrations for each sequence together with its postprocessing steps in the absence of motion. A six‐compartment phantom was prepared in a silicone ice‐cube tray with varying concentrations of Cr (Figure S2). The following were added to each compartment: agar (Sigma‐Aldrich; 2.3% w/w), NaCl (Sigma‐Aldrich; 43 mmol/L), sodium dodecyl sulphate (Sigma‐Aldrich; 43 mmol/L), NiCl_2_ (Sigma‐Aldrich; 0.89 mmol/L), peanut oil (Tesco, UK; 1% v/v) and Cr (Sigma‐Aldrich; 0, 5, 10, 20, 40 and 80 mmol/L). The mixture was heated to boiling point until the agar dissolved completely and the solution turned clear (after around 30 min), and was then left to set overnight.

Spectra were acquired from the phantom from a 20 × 20 × 20 mm^3^ voxel in the centre of each compartment after B_0_ shimming with the vendor's ‘standard’ method over an adjustment volume of 40 × 40 × 40 mm^3^. For each compartment, 30 individual water‐suppressed and three nonwater‐suppressed measurements (i.e. shots) were obtained with STEAM and PRESS using the product WET water suppression and product WET calibration, and then again using WSC water suppression (i.e. all four combinations). For nonwater‐suppressed acquisitions, only the gradients were active in the water‐suppression module (WSC or WET), and not the RF pulses. The acquisition parameters were 1024 points, 2 kHz bandwidth, 4 s TR, 10 ms TE for STEAM and 26 ms TE for PRESS, and 50 Hz water suppression pulse bandwidth. The scanner centre frequency was set at water (4.7 ppm) for nonwater‐suppressed acquisitions and the methyl group in Cr (3.0 ppm) for water‐suppressed acquisitions.

#### 3.2.2 Reproducibility study in healthy volunteers

Ten healthy volunteers (seven males, three females; mean age ± standard deviation [SD] = 29.3 ± 4.0 [range 23–34] years; body mass index [BMI] = 23.7 ± 4.1 kg/m^2^) were recruited for this study and gave informed consent in accordance with local board ethics regulations.

**FIGURE 2:**
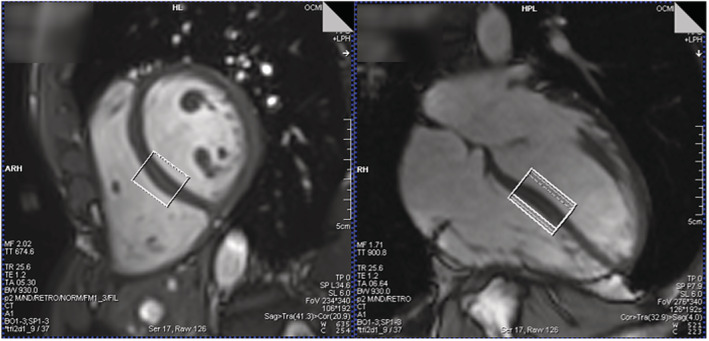
Voxel position (white box) as shown on a short‐axis view (left) and a horizontal long axis view (right)

For each session, the volunteers were positioned head‐first supine in the scanner with ECG gating (Siemens). Cine images were used to obtain horizontal long‐axis and short‐axis views of the heart. The MRS voxel was placed in the midinterventricular septum, to minimise the chance of contamination from epicardial fat. The trigger delay was adjusted for mid‐diastole, where cardiac motion is minimised,^49^ usually around 650 ms for an average R‐R interval of 1000 ms. B_0_ shimming with the vendor's ‘heart’ method was performed over an adjustment volume of 40 × 32 × 30 mm^3^ during a breath‐hold. ^1^H‐MRS data were obtained at end‐expiration from a 32 × 26 × 22 mm^3^ voxel centred on the septum (Figure 2). This voxel contains more ventricular blood compared with previous studies that used smaller voxels (2–8 cm^3^),^50^, ^51^, ^52^, ^53^, ^54^, ^55^, ^56^ but both STEAM and PRESS have been demonstrated to have dark blood properties,^3^, ^50^, ^51^ so contamination from the blood pool should not be significant.^57^

Due to institutional limits on scan durations and the challenge of repeated breath‐holds, it was not possible to run all four sequences. An initial experiment was conducted with six healthy volunteers (three males, three females; age = 32.5 ± 6.2 [range 25–42] years; BMI = 23.1 ± 1.97 kg/m^2^) to compare PRESS‐WET (150 measurements over 30 breath‐holds) with STEAM‐WET (150 measurements over 30 breath‐holds). On the basis of this preliminary study, PRESS‐WET was excluded from the later in vivo comparison (see Figure S2 for spectra and Table S1 for SNR, Cramér‐Rao lower bounds [CRLB] and linewidths for the preliminary study). Thus, in the main study, three sequences were run on the volunteers: STEAM‐WET, STEAM‐WSC and PRESS‐WSC. Parameters for all three sequences matched the phantom experiment, with the following changes: 2 s TR for water‐suppressed acquisitions and the water‐suppression bandwidth for the WSC module was set at 65 Hz. For STEAM‐WET, 150 water‐suppressed spectra were acquired over 30 breath‐holds and three nonwater‐suppressed spectra were acquired over one breath‐hold to give a high‐quality ‘reference’ dataset.

**TABLE 1:** Metabolite concentrations and fat fractions (mean ± SD over all n subjects) are shown in, with the number of outliers in each dataset denoted by the number of ‘^+^’ in brackets, that is, (^+^) means one outlier, (^++^) means two outliers, etc. Any statistically significant changes in metabolite concentration between healthy volunteers and patients as measured by a particular sequence is indicated by * (when *p* < 0.05) or ** (when *p* < 0.01)

Mean ± standard deviation of metabolite concentration					
Group	Sequence	Meas.	Lipid (%)	Creatine (μmol/g)	Choline (μmol/g)
Healthy n = 10	STEAM‐WET‐150	150	0.30 ± 0.15 (^+^)	29.6 ± 7.0 (^++^) *	7.9 ± 6.7
	PRESS‐WSC	60	0.30 ± 0.11 (^+^)	31.5 ± 3.1 (^+^) **	8.3 ± 4.4 (^+^) *
Patients n = 8	STEAM‐WET‐150	150	0.48 ± 0.51 (^+^)	8.0 ± 2.3 (^+^) *	4.6 ± 3.2
	PRESS‐WSC	60	0.25 ± 0.19 (^+^)	9.3 ± 6.0 **	3.9 ± 3.2 *

For STEAM‐WSC and PRESS‐WSC, 60 water‐suppression cycled spectra (i.e. 30 pairs) were acquired over 10 breath‐holds and three water‐nonsuppressed spectra were acquired over one breath‐hold. Across all sequences, each breath‐hold was 11–13 s long, and a 13–20 s pause was given between each breath‐hold. Each session took around 1 h in total.

To obtain repeatability data, each volunteer was scanned in two different sessions on the same day. They were taken out of the scanner between sessions, given a 5‐min break, and then the scan protocol was repeated. The total scan time for each volunteer was just over 2 h including the repeat session. However, the large number of breath‐holds and long scan times were challenging for some volunteers. Based on an interim analysis of data from the first session in the first three volunteers, the STEAM‐WSC was not repeated in the second session for the remaining volunteers.

#### 3.2.3 In vivo study in patients

Thirteen patients (10 males, three females; age = 75.9 ± 6.3 [range 63–83] years; BMI = 27.4 ± 4.3 kg/m^2^) with severe symptomatic aortic or mitral stenosis were recruited as part of a project approved by regional (South Central Oxford C, 16/SC/0323) and local ethics and governance panels. Out of the group, eight patients had aortic stenosis with preserved LVEF, three had aortic stenosis with reduced ejection fraction and one had mitral stenosis. The inclusion criteria were age 18–85 years, with severe aortic stenosis and due to undergo clinically indicated aortic valve replacement, or no pressure‐ or volume‐loading valve disease (i.e. no hypertrophy) but due to undergo cardiac surgery and willing to donate cardiac biopsy. The exclusion criteria were prior myocardial infarction, flow‐limiting coronary disease, more than mild bystander valve disease, renal impairment (estimated glomerular filtration rate < 30 mL/min), or contraindication to MRI scanning. A table summarising the patient demographics is included in the supporting information (Table S2).

For each patient, 150 measurements (30 breath‐holds) were obtained with the STEAM‐WET sequence as the STEAM‐WET‐150 reference dataset and 60 measurements (10 breath‐holds) were obtained with the PRESS‐WSC sequence.

### 3.3 Data analysis

#### 3.3.1 Data postprocessing

Signals from individual coil elements for each shot were combined and averaged using MATLAB (Mathworks, Natick, MA, USA). Weighting and phase‐correction factors for each coil were calculated from the water peak in the nonwater‐suppressed spectra and applied to the water‐suppressed spectra.^3^, ^58^

The spectrum from each transient was phase‐ and frequency‐corrected based on either the lipid peak (for STEAM‐WET and PRESS‐WET) or the residual water peak (STEAM‐WSC and PRESS‐WSC) before averaging. A more detailed explanation of the postprocessing steps for WSC sequences can be found in the Theory section and in Figure 1. All spectra were analysed using the OXSA toolbox.^59^ For the Cr‐fat phantom, five Lorentzian peaks were fitted: residual water around 4.7 ppm, Cr CH_2_ peak around 3.9 ppm, Cr CH_3_ peak around 3.0 ppm and two lipid peaks around 1.28 and 0.84 ppm. For in vivo WET water‐suppressed spectra, residual water signals were removed by the Hankel–Lanczos singular value decomposition (HLSVD) method.^60^, ^61^ The resulting spectra were fitted with seven peaks: residual water, two peaks for the methyl and methylene group of tCr at 3.0 and 3.9 ppm; a peak for the methyl group of Cho derivatives at 3.2 ppm; and three peaks for lipids chains at 1.99 ppm (allylic), 1.28 ppm (methylene) and 0.84 ppm (methyl).^4^ Note that although we fit two peaks for Cr, we used only the Cr CH_3_ signal at 3.0 ppm for analysis of tCr. In this paper, we use ‘choline’ to refer to any trimethylammonium (TMA) compound, as used by Gillinder et al.^6^; however, the peak is likely to include contributions from other nitrogen‐containing compounds such as carnitine or taurine. Each raw transient, intermediate outputs from the postprocessing pipeline, and the final fittings, were all visually inspected for potential errors.

Across all spectra, the SNR of each peak was estimated by taking the ratio of the peak height in a 10‐Hz exponentially apodised spectrum (matched filtering) to the SD of the noise extracted from the Fourier Transform of the last 100 points of the free induction decay of the filtered spectrum.^62^ Note that care must be taken when comparing spectroscopy SNR values between studies because there are many slightly different definitions of SNR. The accuracy of the spectral fitting was estimated by calculating the CRLB.^63^ Due to time constraints, we were unable to run 150 breath‐holds for all methods. Thus, to enable a fair comparison, we also randomly selected 12 breath‐holds from the STEAM‐WET‐150 data to give a 60‐measurement subset. For clarity, we refer to these subsampled data as ‘STEAM‐WET‐60’. This subsampling was performed for STEAM‐WET‐150 data in both healthy volunteers and patients.

#### 3.3.2 Metabolite quantification

Fitted areas for each metabolite A were corrected for T_1_ and T_2_ decay and proton density according to:

(3) $$S_{A}^{*}=S_{A}\times F_{1,A}\times F_{2,A}\times N_{A},$$

(4) F1,A=1−e−TRT1,A−1,

(5) F2,A=eTET2,A,

where S_A_ refers to the uncorrected signal peak integral, S_A_* refers to the corrected signal peak integral, F_1, A_ and F_2, A_ are T_1_ and T_2_ correction factors and *N*_A_ is the proton density.

For the phantom, the T_1_ values were determined to be 0.991 s for the Cr CH_3_ peak and 1.04 s for water, while the T_2_ values were determined to be 321 ms for the Cr CH_3_ peak and 62.7 ms for water. As there are no published values for T_1_ and T_2_ of cardiac metabolites at 3 T, T_1_ and T_2_ values from skeletal muscle were used instead. Myocardial lipid T_1_ and T_2_ values were taken as the average of intramyocellular and extramyocellular lipids. The following T_1_ values were used: 0.459 s for the lipid methylene peak^64^; 1.73 s for the Cr CH_3_ peak^49^; 1.37 s for Cho^49^; and 1.64 s for water.^49^ For T_2_ correction, the following T_2_ values were used: 78 ms for the lipid methylene peak^64^; 177 ms for the Cr CH_3_ peak^49^; 134 ms for Cho^49^; and 35 ms for water.^65^

For in vivo acquisitions, Cr and Cho concentrations were calculated in μmol per gram of wet tissue (μmol/g) according to: $\left[A\right]=S_{A}^{*}÷S_{\text{water}}^{*}\times \left[\text{water}\right]$. The concentration of pure water was taken to be 55.5 mol/L and the myocardial tissue was assumed to contain 72.7% water by weight.^51^ Myocardial lipid content is represented by the corrected ratio of the lipid methylene peak (1.28 ppm) area to the unsuppressed water peak area, expressed as a percentage.^3^

#### 3.3.3 Statistical analysis

Sequence performance was assessed by comparing SNR, CRLB and linewidths of the three main metabolite peaks: lipid methylene peak, Cr CH_3_ peak and Cho peak. The values were described using medians and interquartile ranges, and paired Wilcoxon signed‐rank tests (two‐tailed) were conducted across all possible pairings for SNR and CRLB in healthy volunteers. Metabolite concentrations in both healthy volunteers and patients were described using means and SDs with outliers identified as points more than three scaled median absolute deviations (MAD) away from the median and were excluded from the analysis.

Intersubject coefficients of variation (σ_i_^2^) in metabolites were calculated for healthy volunteers by dividing the SD of the metabolite concentrations by the overall mean. Repeatability of STEAM‐WET and PRESS‐WSC were compared by calculating the intrasubject coefficients of variation (CV) and coefficients of repeatability (CR) for Cr, Cho and lipid quantification. The intrasubject CV can be calculated by taking the ratio of the SD of within‐subject measurements (i.e. the SD of the difference between measurements 1 and 2, also known as σ_w_^2^) to the overall mean.^66^ The CR is the value below which the absolute difference of the two measurements would lie with 95% probability and is calculated by multiplying σ_w_^2^ by 2.77 ( $\sqrt{2}\times 1.96$).^67^

Power calculations were also performed using repeatability data from healthy volunteers with G*Power (version 3.1.9.7; Heinrich Heine Universitat, Dusseldorf, Germany).^68^, ^69^ The two‐tailed a priori power calculation was performed with the following parameters: test family: ‘t tests’; and statistical test: ‘means: difference between two dependent means (matched pairs)’. To calculate the number of subjects needed to determine statistical significance (power = 95%, α = 0.05) for a 20% change in concentration for each metabolite for STEAM‐WET‐150 (150 meas.) and PRESS‐WSC (60 meas.), the ‘mean of difference’ was set to 20% of the mean concentration and the ‘SD of difference’ was set to be the SD of within‐subject measurements (σ_w_^2^).

Lastly, unpaired Student's t‐tests (two‐tailed, equal variance assumed) were conducted to analyse the differences in metabolite concentrations between healthy volunteers and patients. The metabolite concentrations in healthy volunteers were averaged across both sessions.

## 4 RESULTS

### 4.1 Phantom study

**FIGURE 3:**
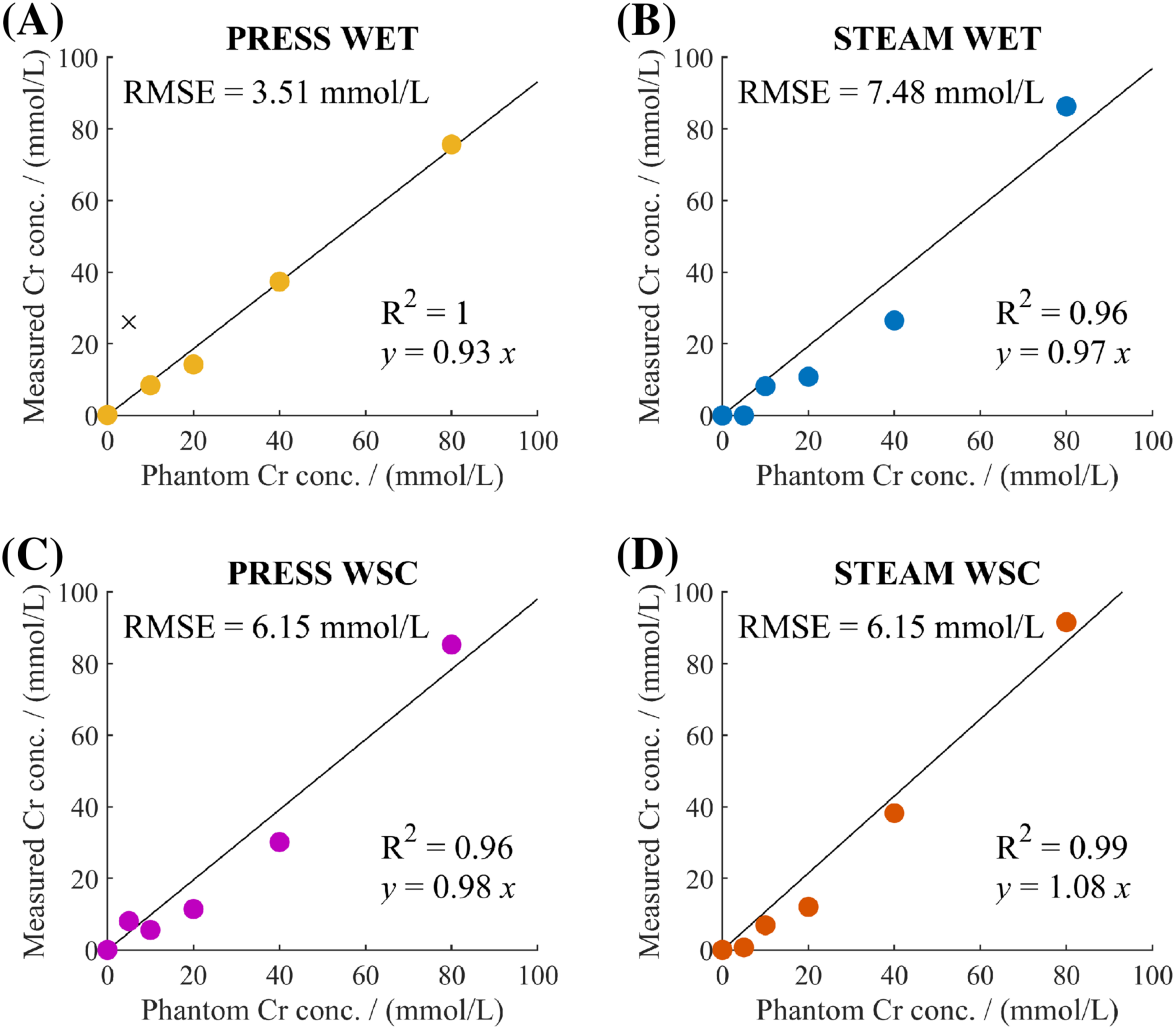
Correlation of measured creatine (Cr) concentration and the actual Cr concentration in each phantom compartment measured with A, PRESS‐WSC, B, STEAM‐WET, C, PRESS‐WSC and D, STEAM‐WSC. An anomalous point in A has been identified and labelled using a black cross. This point was subsequently excluded from data analysis. RMSE, root‐mean‐squared error

The correlation between the measured and actual Cr concentration in the phantom for the four sequences is shown in Figure 3. An anomalous point was identified for PRESS‐WET and was subsequently excluded. All correlations had R^2^ ≥ 0.96 and the slopes of the linear regression lines (β) ranged from 0.93 to 1.08. The root‐mean‐squared errors (RMSEs) in concentration measurements for each sequence were 3.51 mmol/L for PRESS‐WET, 7.48 mmol/L for STEAM‐WET, 6.15 mmol/L for PRESS‐WSC and 6.15 mmol/L for STEAM‐WSC. SNR and CRLB values are shown in Table S2.

### 4.2 Sequence comparison in healthy volunteers

**FIGURE 4:**
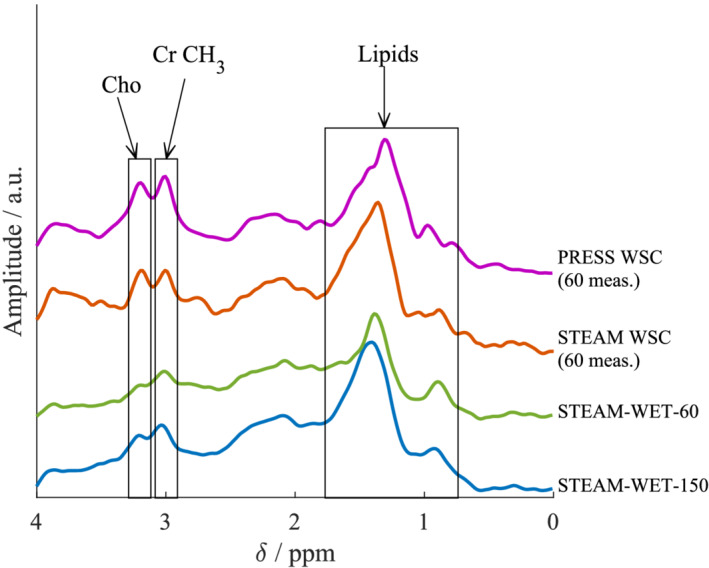
Spectra from a single volunteer comparing all four protocols tested in the volunteer study

Figure 4 shows the four spectra acquired from one healthy volunteer: STEAM‐WET‐150, STEAM‐WET‐60, STEAM‐WSC and PRESS‐WSC.

**FIGURE 5:**
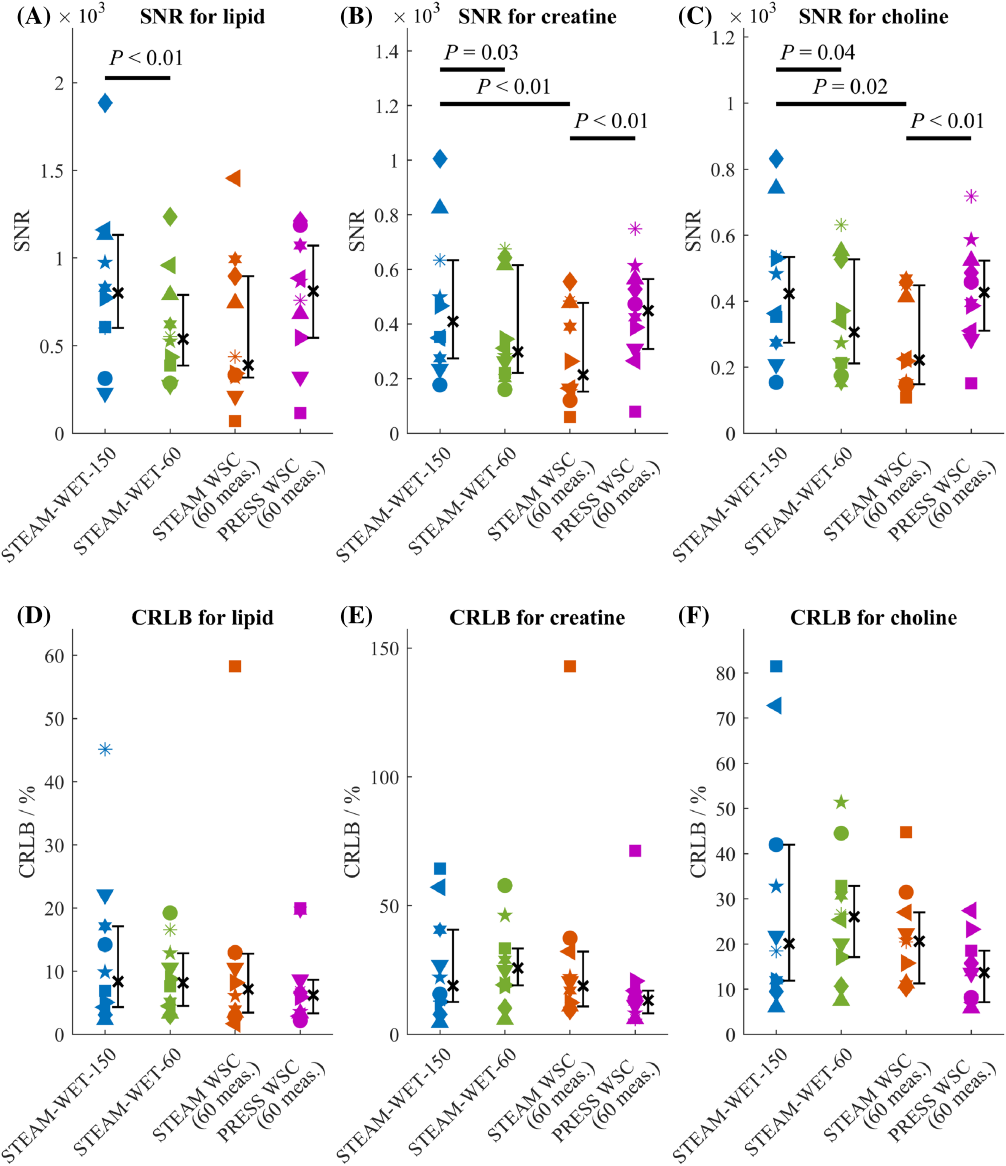
Comparision of Cramér‐Rao lower bounds (CRLB) and signal‐to‐noise ratio (SNR) across all four protocols tested in the volunteer study for A and D, lipids, B and E, creatine and C and F, choline. The black cross and the attached whiskers represent the median and the interquartile across all healthy volunteers. For each plot, Wilcoxon signed‐rank tests were conducted across all six possible pairs and all significant *p*‐values (*p* ≤ 0.05) are shown

SNR and CRLB values are shown in Figure 5 for healthy volunteers. The metabolite concentrations for all healthy volunteers and patients are presented in Table 1, while SNR, CRLB and linewidths are presented in Table S3. The SNR for all three metabolites decreased between STEAM‐WET‐150 and STEAM‐WET‐60 with *p‐*values of less than 0.01 for lipid, less than 0.03 for Cr and less than 0.04 for Cho. On the other hand, despite being acquired in less than half the scan time, PRESS‐WSC showed no significant decrease in SNR for all peaks compared with STEAM‐WET‐150. The CRLB values also followed these trends. CRLB increased (i.e. there was worse fit precision) when the number of measurements was decreased from STEAM‐WET‐150 to STEAM‐WET‐60 as expected. Yet with only 60 measurements, the PRESS‐WSC sequence gave the lowest CRLB values (best fit precision) for all metabolites.

Lastly, randomisation of the choice of breath‐holds for STEAM‐WET‐60 did not have any significant effects on SNR, CRLB or linewidths (Figure S4).

### 4.3 Metabolite concentrations in healthy volunteers

**FIGURE 6:**
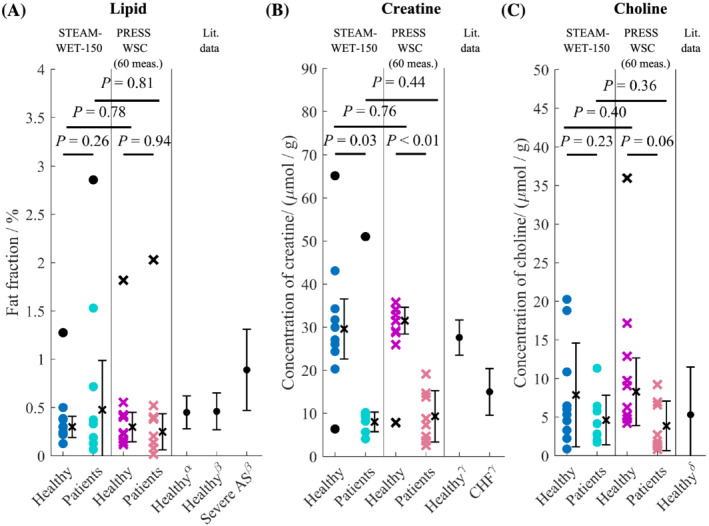
Comparison of metabolite concentrations and fat fractions in healthy and patient cohort with STEAM‐WET‐150 (‘o’) and PRESS‐WSC (‘×’) for A, lipids, B, creatine and C, choline against various published literature values. For healthy volunteers, the values are an average between scan 1 and scan 2 while literature data are taken from the following sources: Rial et al. (α),^3^ Mahmod et al. (β),^73^ Nakae et al. (γ)^50^ and Gilinder et al. (δ).^6^ Data from Rial et al. and Mahmod et al. were obtained at 3 T, while Nakae et al. and Gilinder et al. obtained their data at 1.5 T. For the current study, data points more than 1.5 interquartile ranges above the upper quartile or below the lower quartile are treated as outliers (denoted in black). The black cross and the attached whiskers represent the mean and the standard deviations across all healthy volunteers. The *p*‐values from the unpaired Student's t‐tests are also provided. AS, aortic stenosis; CHF, chronic heart failure

Myocardial metabolite concentrations in both healthy volunteers and the patient group are shown in Figure 6. In healthy volunteers, the average fat fraction measured with STEAM‐WET‐150 was 0.30% ± 0.11%, and with PRESS‐WSC it was 0.30% ± 0.15% (*p =* 0.78); the average Cr concentration measured with STEAM‐WET‐150 was 29.6 ± 7.0 μmol/g, and with PRESS‐WSC it was 31.5 ± 3.1 μmol/g (*p* = 0.76); and the average Cho concentration measure with STEAM‐WET‐150 was 7.9 ± 6.7 μmol/g, and with PRESS‐WSC it was 8.3 ± 4.4 μmol/g (*p* = 0.40). There were no significant differences in any of the reported fat fractions or metabolite concentrations between the two sequences.

### 4.4 Repeatability in healthy volunteers

**FIGURE 7:**
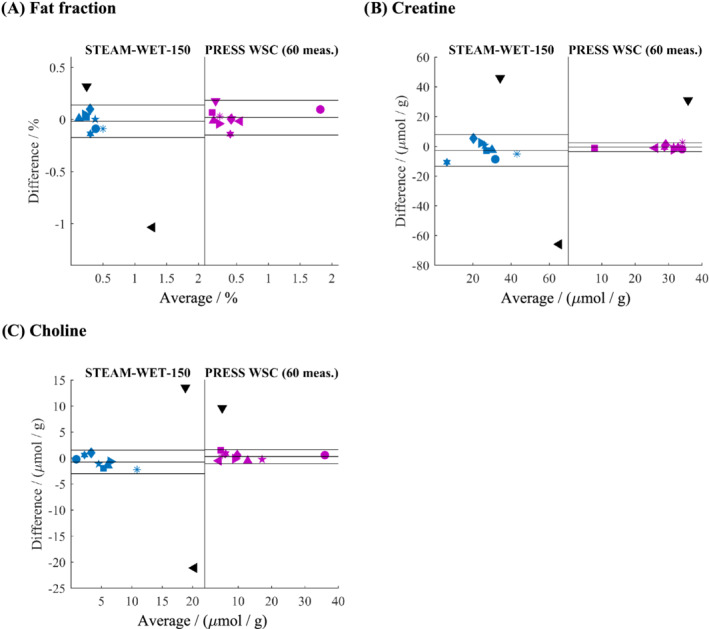
Bland–Altman plots: A, for lipids with STEAM‐WET‐150 (coefficient of repeatability [CR] = 0.22%) and PRESS‐WSC (CR = 0.24%); B, for creatine with STEAM‐WET‐150 (CR = 15.1 μmol/g) and PRESS‐WSC (CR = 4.19 μmol/g); and C, for choline with STEAM‐WET‐150 (CR = 3.2 μmol/g) and PRESS‐WSC (CR = 1.92 μmol/g). The mean difference and the limits of agreement are shown. Data points more than 1.5 interquartile ranges above the upper quartile or below the lower quartile are treated as outliers (coloured black)

Repeatability results are shown in Table 2 and Bland–Altman plots in Figure 7. For lipid quantification, the CR values are comparable for both sequences, with CR being 0.22% for STEAM‐WET‐150 and 0.24% for PRESS‐WSC (60 meas.). For both Cr and Cho, PRESS‐WSC showed improved performance. The CR for Cr was 4.19 μmol/g for PRESS‐WSC and 15.1 μmol/g for STEAM‐WET‐150. A similar improvement was seen for Cho, with CR being 1.92 μmol/g for PRESS‐WSC and 3.2 μmol/g for STEAM‐WET‐150.

**TABLE 2:** Intersubject coefficient of variation (σ_i_^2^), intrasubject coefficient of variation (σ_w_^2^) and coefficient of repeatability for lipids, creatine and choline quantification in healthy volunteers (n = 10)

Repeatability data in healthy volunteers (n = 10)					
	Sequence	Meas.	Lipid	Creatine	Choline
Intersubject coefficient of variation (σ_i_^2^)	STEAM‐WET‐150	150	37%	24%	86%
	PRESS‐WSC	60	52%	10%	53%
Intrasubject coefficient of variation (σ_w_^2^)	STEAM‐WET‐150	150	26%	21%	23%
	PRESS‐WSC	60	19%	5%	6%
Coefficient of repeatability	STEAM‐WET‐150	150	0.22%	15.1 μmol/g	3.20 μmol/g
	PRESS‐WSC	60	0.24%	4.19 μmol/g	1.92 μmol/g

As an illustration of the power of 3‐T ^1^H‐MRS to assess Cr and/or Cho in clinical studies, Table 3 compares the calculated sample sizes needed to detect a 20% change in metabolite levels for paired and independent studies for both sequences. Sample sizes for independent studies are reported as the numbers of participants per group, assuming that only 60% of subjects have quantifiable spectra.

**TABLE 3:** Power calculations shows the number of subjects (N) needed to determine statistical significance (power = 95%, α = 0.05) for a 20% change in concentration for each metabolite using STEAM‐WET‐150 (150 measurements) and PRESS‐WSC (60 measurements). The calculations assume that only 60% of the subjects have quantifiable spectra

	STEAM‐WET‐150	PRESS‐WSC
No. measurements	150	60
Scan duration	~30 min	~10 min
Numbers needed for paired studies assuming 60% ‘success’ rate in acquisition		
power = 95%, α = 0.05		
Lipid	42	49
Creatine	24	7
Choline	17	9

### 4.5 Demonstration in patient cohort

All patients completed the examination. In one patient, the number of measurements per breath‐hold had to be reduced to four to shorten the length of the breath‐hold. Five patient datasets were discarded as they either had fitted linewidths of less than 2 Hz for any of the metabolites, which indicates that there was only noise in the relevant spectral region, or because of a CRLB value for the lipid peak of more than 500% in either of the acquisitions. All five had poor STEAM‐WET data; four out of five also had poor PRESS‐WSC data.

**FIGURE 8:**
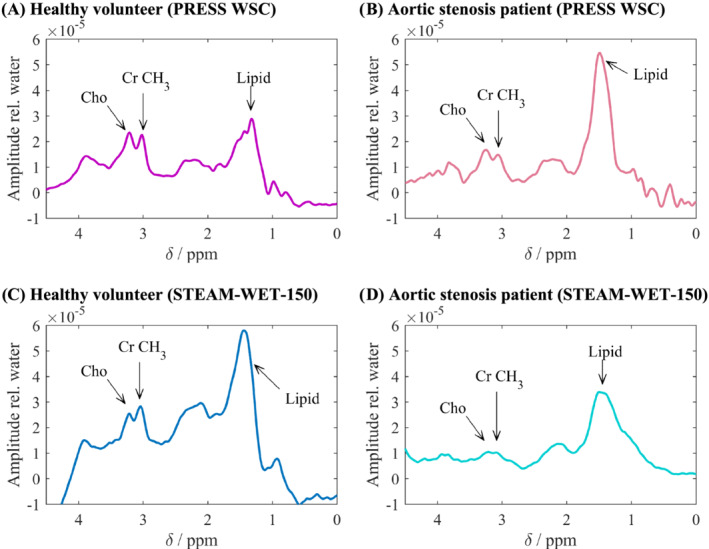
Spectra from a healthy volunteer (left, both A and C) compared with that of a patient (right, both B and D) suffering from aortic stenosis. The spectra were acquired with PRESS‐WSC in a healthy volunteer (A) and a patient (B), and with STEAM‐WET‐150 in a healthy volunteer (C) and a patient (D). All spectra are scaled by the corresponding water peak amplitude. A decrease in creatine (Cr) is seen between these pairs

The remaining eight datasets consisted of seven patients with aortic stenosis with preserved systolic function and one patient suffering from aortic stenosis with impaired systolic function. Figure 8 shows spectra from a patient compared with those from a healthy volunteer.

For the patient cohort, the average fat fractions were 0.48% ± 0.51% when measured with STEAM‐WET‐150, and 0.25% ± 0.19% when measured with PRESS‐WSC; average Cr concentrations were 8.0 ± 2.3 μmol/g when measured with STEAM‐WET‐150, and 9.3 ± 6.0 μmol/g when measured with PRESS‐WSC; while average Cho concentrations were 4.6 ± 3.2 μmol/g when measured with STEAM‐WET‐150, and 3.9 ± 3.2 μmol/g when measured with PRESS‐WSC. There were no significant differences in the reported fat fraction or metabolite concentrations between the two sequences.

When comparing the patient cohort with healthy volunteers, there were no significant changes in lipid levels for both sequences (*p* = 0.26 for STEAM‐WET‐150 and *p* = 0.94 for PRESS‐WSC), but significant decreases in Cr levels were observed in the patient group compared with healthy volunteers for both sequences (*p* = 0.03 for STEAM‐WET‐150 and *p* < 0.01 for PRESS‐WSC). The average Cho levels were also lower in the patient group than in healthy volunteers but these changes were not significant (*p* = 0.23 for STEAM‐WET‐150 and *p* = 0.06 for PRESS‐WSC).

## 5 DISCUSSION

### 5.1 Sequence performance

#### 5.1.1 Phantom

Our phantom results show that all four sequences have the potential to quantify Cr levels in the heart in vivo.

#### 5.1.2 Healthy volunteers

For in vivo acquisitions in healthy volunteers, it would have been ideal to compare STEAM‐WET, PRESS‐WET, STEAM‐WSC and PRESS‐WSC in a single study with sufficient measurements to get a robust quantitation from all methods. However, due to institutional limits on the duration of a scan, this was not feasible. Thus, PRESS‐WET was excluded based on the results of a preliminary study that compared STEAM‐WET and PRESS‐WET in six healthy volunteers, where it showed inferior SNR, CRLB and linewidths in all three metabolites.

In the main in vivo study, PRESS‐WSC showed increased fitting accuracy (i.e. lower CRLB values) and improved repeatability compared with the longer ‘gold standard’ STEAM‐WET‐150 approach, despite having less than half the number of measurements. These improvements translate to decreases in the number of subjects that would be needed to detect a specified biological effect on Cr or Cho metabolism, as shown by the power calculations in Table 3. They also indicate that PRESS‐WSC may be a shorter (and hence more patient‐friendly) alternative to STEAM‐WET for cardiac ^1^H‐MRS.

PRESS‐WSC also outperformed both STEAM‐WET and STEAM‐WSC in terms of the raw SNR for matched scan durations. It is tempting to attribute the improvement in SNR seen in PRESS‐WSC to the difference in signal intensity between PRESS and STEAM. PRESS acquires a spin‐echo signal that inherently has twice the signal intensity compared with a stimulated echo signal acquired by STEAM and this is clearly illustrated in the phantom dataset. However, perhaps surprisingly, PRESS‐WET showed inferior performance in both SNR and CRLB compared with STEAM‐WET in the preliminary in vivo study (Figure S3). This indicates that SNR is controlled by other factors in vivo, for example, differences in sensitivity to motion and diffusion effects between the two SVS sequences.

In terms of linewidths, both WSC sequences showed decreases in metabolite linewidths compared with STEAM‐WET‐150, which could be due to more accurate frequency and phase corrections based on the larger, narrower residual water peak, rather than a smaller, broader lipid peak. However, the linewidths achieved using STEAM‐WSC sequence are on average lower compared with PRESS‐WSC. This could be considered as surprising because PRESS‐WSC was regarded as superior to STEAM‐WSC when assessed with other measures of spectral quality (e.g. SNR, CRLB and repeatability). We observed a similar trend between STEAM‐WET and PRESS‐WET in our preliminary experiment, thus, we hypothesise that the difference in linewidth could be due to the differences in the STEAM and PRESS sequences. For example, PRESS had a longer TE, which would increase sensitivity to small motion effects, and PRESS has 180° pulses that may show more slice edge effects than the 90° pulses in STEAM (with a consequent increase in true voxel size, and hence in linewidth). There are also more complex factors in vivo that might have contributed to the final differences, such as bulk motion and diffusion effects, which will need to be further investigated to fully understand this surprising trend.

Across all in vivo acquisitions, the bandwidth of water‐suppression pulses in WSC was larger (65 Hz or 0.5 ppm) than that for WET (50 Hz or 0.4 ppm). This slightly larger bandwidth was determined through empirical observation in a series of pilot experiments. We suspect that the need for increased water‐suppression bandwidth arises because the WSC module needs to either invert or not invert the water. If the water peak drifts in some transients out of the water‐suppression bandwidth, then this can lead to distorted lineshapes that are not fully cancelled during the WSC postprocessing.

This increased water‐suppression bandwidth means that care should be taken when quantifying metabolites with peaks close to water (e.g. the methylene peak of Cr at 3.9 ppm). It is possible that those peaks might be partially suppressed in the presence of severe shot‐to‐shot frequency drifts. In the current study, to avoid this potential issue, we only used the peak amplitude of the Cr methyl group at 3.0 ppm for cardiac Cr quantification, and not the Cr methylene peak at 3.9 ppm. Furthermore, we note that there is no significant difference in the closer Cho signal at 3.2 ppm between PRESS‐WSC and STEAM‐WET in our study, suggesting that this effect is not of concern here.

Another possible factor that affects metabolite quantification is the lack of preparation scans in our protocol. Although preparation scans ensure that a steady state is reached before signal acquisition, they also lengthen the duration of each breath‐hold. A quick calculation using literature T_1_ values and nominal flip angles shows that the error in quantification due to the slightly higher signal in the first transient is very small, accounting for percentage differences smaller than 0.75% for Cr CH_3_ and 2.2% for Cho. These potential errors are modest compared with other sources of error in the fitted amplitudes and can be neglected.

#### 5.1.3 Patient cohort

In the patient cohort, PRESS‐WSC also showed comparable fitting accuracy compared with STEAM‐WET‐150. In just 60 measurements, PRESS‐WSC showed an average increase of a mere 2% in CRLB values compared with 150 measurements of STEAM‐WET‐150 (averaging for lipids, Cr and Cho). The benefits of using PRESS‐WSC over STEAM‐WET become more obvious when comparing PRESS‐WSC with STEAM‐WET‐60, where PRESS‐WSC delivered an average decrease of 28% in CRLB values and of 18% in linewidths.

STEAM‐WET‐150 and PRESS‐WSC both gave spectra with acceptable quality in all healthy volunteer scans. Yet, in patients, STEAM‐WET‐150 only gave acceptable spectra for quantitative analysis in eight out of the 13 patients scanned. PRESS‐WSC made a small improvement in this respect with one more patient having analysable data quality despite the much reduced scan duration. Nevertheless, we are continuing to explore ways to achieve full robustness in patients as well as in healthy volunteers. We believe the underlying issues are not sequence‐specific but include the familiar issues of heart rate variability, body habitus, compliance with breath holding, age and MR scan familiarity, etc.

#### 5.1.4 Repeatability

We showed that in less than half the scan duration time (60 vs 150 measurements, or 30 vs 10 breath‐holds), PRESS‐WSC greatly improves intrasubject repeatability for the quantification of Cr and Cho, and that it maintains repeatability for lipids (Figure 7) compared with STEAM‐WET.

For the repeatability of lipid quantification, Rial et al. reported an intrasubject CV of 19% using a STEAM sequence with breath‐holding at 3 T.^3^ There have also been a number of previous studies published at 1.5 T using gating methods. Szczepaniak et al. reported an intrasubject CV of 19% using a respiratory gating,^70^ and Felblinger et al. reported an intrasubject CV of 13% using a double cardiac and respiratory gating method.^71^ Our results for PRESS‐WSC in healthy volunteers (i.e. an intrasubject CV of 19%) are in good agreement with these reports.

Felblinger et al. also reported intersubject and intrasubject variabilities in cardiac concentrations of Cr and Cho (labelled there as ‘TMA’). In their study, using a double‐triggered PRESS sequence at 1.5 T, they found the intersubject/intrasubject variability for Cr to be 16%/10% and for Cho to be 56%/9%.^71^ Similarly, Nakae et al. reported a CV of 7.4% in repeated Cr measurements of eight volunteers using a free breathing PRESS sequence at 1.5 T with cardiac gating.^72^ These values are similar to ours for PRESS‐WSC; however, our STEAM‐WET‐150 protocol yielded higher intersubject/intrasubject variability values for both metabolites.

From both the Bland–Altman plots (Figure 7) and the CR, it can be seen that the lipid repeatability is differently influenced compared with Cr and Cho. Possible explanations for this include that the lipid peak is influenced by J‐coupling. The difference in J‐coupling evolution with different TEs could account for some of the difference in the repeatability changes. Alternatively, because the heart is surrounded by a layer of epicardial fat, inconsistent diaphragm positions between breath‐holds and patient motion could perhaps result in some spectral transients being contaminated by epicardial fat.

#### 5.1.5 Simplicity of implementation

We note in passing that many of the prior studies in cardiac Cr and Cho quantitation have used relatively complicated motion compensation strategies. Our approach has a benefit that it is straightforward to implement because the only sequence modifications are small alterations to the water suppression module. Other changes are implemented in off‐scanner reconstruction tools, which are simpler to alter. Therefore, even where our repeatability is comparable with some prior studies, this simplicity of implementation is a pragmatic benefit of the WSC approach.

### 5.2 Metabolite quantification and changes in patient cohort

In both healthy volunteers and patients, the measured fat fractions and metabolite concentrations did not differ significantly between STEAM‐WET and PRESS‐WSC. Both sequences also gave absolute myocardial Cr and Cho concentrations and fat fractions that were similar to values from the other sequences and from previous studies.^4^, ^6^, ^50^, ^51^, ^71^

There was no significant difference in myocardial lipid concentrations between patients and controls. The results were 0.30% ± 0.11% in healthy controls and 0.48% ± 0.51% in patients when measured with STEAM‐WET‐150 (*p* = 0.26), and 0.30% ± 0.15% in healthy controls and 0.25% ± 0.19% in patients when measured with PRESS‐WSC (*p* = 0.94). This was unexpected,^73^ however, Marfella et al.^74^ reported that lipid contents are significantly higher in aortic stenosis patients with metabolic syndromes than in those without metabolic syndromes. Although none of our patients had diabetes mellitus, insulin resistance is a known correlate of heart failure,^75^ so it is likely that our cohort consisted of a mixture of the two groups, which would be expected to attenuate differences in observed lipid concentration vs healthy volunteers.

There are few reports on myocardial Cr levels in heart disease. For healthy myocardium, Nakae et al. reported Cr concentrations measured at 1.5 T to be 27.6 ± 4.1 μmol/g^51^ and Bottomley et al. reported 28 ± 6 μmol/g.^76^ This is in close agreement with the values we measured in healthy volunteers (29.6 ± 7.0 μmol/g when measured with STEAM‐WET‐150 and 31.5 ± 3.1 μmol/g when measured with PRESS‐WSC). Nakae et al. later studied patients suffering from congestive heart failure due to dilated or hypertrophic cardiomyopathy and found a reduction in myocardial Cr concentrations of 12.5 μmol/g^50^ (i.e. a 15.5 μmol/g or 45% reduction). The CR of Cr concentration measured with our PRESS‐WSC (60 meas. over 10 breath‐holds, CR = 4.19 μmol/g) is roughly one third of this reduction, while that of STEAM‐WET‐150 (CR = 15.1 μmol/g) is comparable with the reduction, suggesting that the PRESS‐WSC approach is better suited to assessing Cr depletion. In our patient cohort, we observed a significant decrease in Cr concentrations in patients vs volunteers with an average reduction of 21.9 μmol/g across the two sequences. Despite the small cohort scanned in this study, these findings suggest that PRESS‐WSC, with its improved repeatability, is a better choice for assessing biologically relevant changes in Cr concentration than STEAM‐WET.

In a study conducted at 1.5 T by Gillinder et al., the myocardial Cho water ratio was 0.24% ± 0.28% before any corrections.^6^ By correcting for relaxation and proton density, this result of Gillinder et al. corresponds to a myocardial Cho concentration of 9.6 ± 11.1 μmol/g, which is in line with our findings of 7.9 ± 6.7 μmol/g with STEAM‐WET‐150 and 8.3 ± 4.4 μmol/g with PRESS‐WSC. Fillmer et al. have also recently quantified TMA‐containing compounds in the myocardium at 3 T (i.e. what we refer to as ‘choline’). They reported a mean TMA to Cr ratio of 0.79 before any corrections.^8^ After correcting for relaxation and proton density, the Cho to Cr ratio (S_Cho_*/S_Cr_*) reported by them would be 0.21, which again is similar to our results of 0.27 for STEAM‐WET‐150 and 0.26 for PRESS‐WSC.

We compare total Cho content in patients with aortic stenosis vs volunteers for the first time. The small sample size in the study means that it is impossible to draw any statistically significant conclusions about the variations of total Cho between healthy and patient cohorts at this point, but our work demonstrates the feasibility of ^1^H‐MRS as a clinical tool with which to relate variations in myocardial Cho concentration with variations in myocardial performance.

Recently, a number of studies have linked dietary Cho intake to cardiovascular diseases in animal models.^77^ Human studies to investigate this are more complex due to the significant influence of the intestinal microflora^78^; there is no general agreement yet on the influence of dietary Cho intake on the incidence of cardiovascular diseases.^79^, ^80^, ^81^, ^82^, ^83^ However, preclinical evidence suggests that Cho given as an injection has cardioprotective effects in rat models, including reducing postischaemia reperfusion injuries^84^ and slowing the progression of hypertension,^85^ and the whole‐blood Cho level has been identified as a promising biomarker in acute coronary syndrome.^86^, ^87^ However, most of these studies focused on dietary Cho intake and/or supplements^79^ without measuring any direct changes in Cho concentration in the heart. With emerging research aiming to modulate the cholinergic system and thereby target heart disease,^88^, ^89^ ^1^H‐MRS is a valuable tool for direct measurement of myocardial Cho in vivo.

### 5.3 Future works

In our analysis, we used relaxation times measured in skeletal muscles for quantifying cardiac metabolites. Although a previous imaging study has shown that T_1_ and T_2_ values for heart and skeletal muscles are very similar at both 1.5 and 3 T,^90^ we believe that the accuracy in metabolite quantification could be improved by using relaxation values measured directly in the human heart at 3 T.

We also noted that the performance gains for PRESS‐WSC were more modest in our patient cohort than in volunteers. In a future study, we intend to further optimise the WSC module. For example, it may be advantageous to simply cycle the water signal by 180° on alternate transients without adding the other water‐suppression pulses in what would be a purely ‘water‐cycled’ MRS method analogous to ‘metabolite‐cycled’ methods. It could also be possible to reduce the spacing between water‐suppression pulses or to remove the first 90° pulse in the WSC module to make the PRESS‐WSC acquisition fit more easily into the shorter diastole seen in high heart‐rate, arrhythmic patients, like those we scanned in the current study.

We observed decreases in both Cr and Cho levels in the patient group compared with healthy volunteers. However, we only had a limited study size to provide pathophysiology‐related conclusions. Future studies including more patients with aortic or mitral stenosis are required to gain a better insight into the biochemical changes in the myocardium associated with this diseased state.

## 6 CONCLUSIONS

We have shown that a water‐suppression cycled PRESS sequence improves performance and repeatability in measuring cardiac metabolites (lipids, Cr, Cho) relative to the vendor's standard STEAM and PRESS sequences at 3 T. We quantified cardiac Cr and Cho in a 10–breath‐hold protocol, which was robust in healthy volunteers. Lastly, in patients with severe symptomatic valvular heart disease listed for clinically indicated valve replacement surgery, we were able to detect changes in cardiac metabolites (Cr, Cho, lipids) in a third of the time using PRESS‐WSC compared with the vendor's STEAM‐WET sequence.

## Supporting information


**Figure S1:** Simulation workflow and results showing that amplitudes of metabolite peaks remain unchanged by the weighting factor. The scatter plot at the bottom shows the mean and standard deviation in percentage difference across all 200 sets for each metabolite and each w factor and a reference set (where only the noise varied).
Figure S2: Six compartment phantom in silicone ice cube tray with six different concentrations of creatine (0, 5, 10, 20, 40, and 80 mmol/L). Each compartment is roughly 4.8 × 4.8 × 4.8 cm^3^.
Figure S3: Cardiac spectra of a healthy volunteer obtained by PRESS‐WET (green) and STEAM‐WET (blue) showing the various metabolite peaks: CH_2_ of total creatine (Cr CH_2_), choline (Cho), CH_3_ of total creatine (Cr CH_3_) and lipid peaks. On average across six healthy volunteers, PRESS‐WET had 23% smaller SNR and 88% larger CRLB value for the Cr CH_3_ peak compared to STEAM‐WET.
Figure S4: SNR (A), CRLB (B) and linewidths (C) for the first (red), last (blue) and 3 random sets (green) of 12 breathholds obtained from the STEAM‐WET (150 meas.) acquisition in healthy volunteers. The random set corresponding to the data set used in the paper is labelled as ‘Random 1’ and plotted in a darker shade of green. Each volunteer is represented by a different symbol and data points more than 1.5 interquartile ranges above the upper quartile or below the lower quartile are treated as outliers (coloured black). The black cross and corresponding whiskers show the mean and standard deviation for each data set. No significant difference was found between any of the parameters in the various sets of 12 breathholds.
Table S1: Median and IQR of (A) SNR, (B) CRLB and (C) linewidths of fitted peaks in the STEAM‐WET vs PRESS‐WET pre‐study pilot experiment involving 6 healthy volunteers.
Table S2: Table summarising patient demographics. Values are given as number(%), mean (SD) or median (quartile 1 – quartile 3).
Table S3: SNR and CRLB of each metabolite peak in the phantom. The measurements were obtained from the compartment containing 80 mmol/L of Cr. All acquisition parameters were as described in the Methods section of the paper, except a TE of 40 ms was used for all protocols to ensure a fairer comparison of SNR
Table S4: Medians along with interquartile ranges (IQR) of SNR, CRLB and linewidths over all n subjects are shown in (A), (B) and (C) respectively.

## ENDNOTE

* The peak we refer to as ‘choline’ for brevity may also contain taurine and carnitine. It is denoted by some authors as the ‘trimethylammonium (TMA) containing compounds’ peak.

## References

[1] Goldberg IJ , Trent CM , Schulze PC . Lipid metabolism and toxicity in the heart. Cell Metab. 2012;15(6):805‐812. 22682221 10.1016/j.cmet.2012.04.006 PMC3387529

[2] Boudina S , Abel ED . Diabetic cardiomyopathy revisited. Circulation. 2007;115(25):3213‐3223. 17592090 10.1161/CIRCULATIONAHA.106.679597

[3] Rial B , Robson MD , Neubauer S , Schneider JE . Rapid quantification of myocardial lipid content in humans using single breath‐hold 1H MRS at 3 Tesla. Magn Reson Med. 2011;66(3):619‐624. 21721038 10.1002/mrm.23011 PMC3427889

[4] Faller KME , Lygate CA , Neubauer S , Schneider JE . (1)H‐MR spectroscopy for analysis of cardiac lipid and creatine metabolism. Heart Fail Rev. 2013;18(5):657‐668. 22945240 10.1007/s10741-012-9341-z PMC3782658

[5] Gastl M , Peereboom SM , Gotschy A , et al. Myocardial triglycerides in cardiac amyloidosis assessed by proton cardiovascular magnetic resonance spectroscopy. J Cardiovasc Magn Reson. 2019;21(1):10. 10.1186/s12968-019-0519-6 30700314 PMC6354424

[6] Gillinder L , Goo SY , Cowin G , et al. Quantification of intramyocardial metabolites by proton magnetic resonance spectroscopy. Front Cardiovasc Med. 2015;2. 2:24 10.3389/fcvm.2015.00024 PMC4671339 26664896

[7] Peereboom SM , Gastl M , Fuetterer M , Kozerke S . Navigator‐free metabolite‐cycled proton spectroscopy of the heart. Magn Reson Med. 2020;83(3):795‐805. 31448841 10.1002/mrm.27961

[8] Fillmer A , Hock A , Cameron D , Henning A . Non‐water‐suppressed1H MR spectroscopy with orientational prior knowledge shows potential for separating intra‐ and extramyocellular lipid signals in human myocardium. Sci Rep. 2017;7(1):16898. 29203776 10.1038/s41598-017-16318-0 PMC5714998

[9] Neubauer S . The failing heart — an engine out of fuel. N Engl J Med. 2007;356(11):1140‐1151. 17360992 10.1056/NEJMra063052

[10] Weiss K , Bottomley PA , Weiss RG . On the theoretical limits of detecting cyclic changes in cardiac high‐energy phosphates and creatine kinase reaction kinetics using in vivo ^31^P MRS. NMR Biomed. 2015;28(6):694‐705. 25914379 10.1002/nbm.3302 PMC4433167

[11] Bottomley PA . MRS Studies of Creatine Kinase Metabolism in Human Heart. In: EMagRes. Chichester, UK: John Wiley & Sons, Ltd; 2016:1183‐1202.

[12] Ingwall JS . Weiss RG. Is the failing heart energy starved? On using chemical energy to support cardiac function. Circ Res. 2004;95(2):135‐145. 15271865 10.1161/01.RES.0000137170.41939.d9

[13] Ingwall JS , Kramer MF , Fifer MA , et al. The creatine kinase system in normal and diseased human myocardium. N Engl J Med. 1985;313(17):1050‐1054. 2931604 10.1056/NEJM198510243131704

[14] Bottomley PA . NMR Spectroscopy of the Human Heart. In: Grant DM , Harris RK , eds. Encyclopedia of Nuclear Magnetic Resonance, volume 1: Historical Perspectives. Chichester, UK: Wiley; 2007.

[15] Bottomley PA , Weiss RG . Noninvasive localized MR quantification of creatine kinase metabolites in normal and infarcted canine myocardium. Radiology. 2001;219(2):411‐418. 11323465 10.1148/radiology.219.2.r01ma39411

[16] Valkovič L , Clarke WT , Schmid AI , et al. Measuring inorganic phosphate and intracellular pH in the healthy and hypertrophic cardiomyopathy hearts by in vivo 7T 31P‐cardiovascular magnetic resonance spectroscopy. J Cardiovasc Magn Reson. 2019;21(1):19. 30871562 10.1186/s12968-019-0529-4 PMC6419336

[17] Tian R , Ingwall JS . Energetic basis for reduced contractile reserve in isolated rat hearts. Am J Physiol. 1996;270(4 Pt 2):H1207‐H1216. 8967358 10.1152/ajpheart.1996.270.4.H1207

[18] Gabr RE , El‐Sharkawy A‐MM , Schär M , et al. Cardiac work is related to creatine kinase energy supply in human heart failure: a cardiovascular magnetic resonance spectroscopy study. J Cardiovasc Magn Reson. 2018;20(1):81. 30526611 10.1186/s12968-018-0491-6 PMC6287363

[19] Martin C , Schulz R , Rose J , Heusch G . Inorganic phosphate content and free energy change of ATP hydrolysis in regional short‐term hibernating myocardium. Cardiovasc Res. 1998;39(2):318‐326. 9798517 10.1016/s0008-6363(98)00086-8

[20] Schwarzer M , Faerber G , Rueckauer T , et al. The metabolic modulators, Etomoxir and NVP‐LAB121, fail to reverse pressure overload induced heart failure in vivo. Basic Res Cardiol. 2009;104(5):547‐557. 19294446 10.1007/s00395-009-0015-5

[21] Lionetti V , Stanley WC , Recchia FA . Modulating fatty acid oxidation in heart failure. Cardiovasc Res. 2011;90(2):202‐209. 21289012 10.1093/cvr/cvr038 PMC3078800

[22] Zeisel SH , da Costa K‐AA . Choline: an essential nutrient for public health. Nutr Rev. 2009;67(11):615‐623. 19906248 10.1111/j.1753-4887.2009.00246.x PMC2782876

[23] Gordan R , Gwathmey JK , Xie L‐H . Autonomic and endocrine control of cardiovascular function. World J Cardiol. 2015;7(4):204‐214. 25914789 10.4330/wjc.v7.i4.204 PMC4404375

[24] Mufson EJ , Counts SE , Perez SE , Ginsberg SD . Cholinergic system during the progression of Alzheimer's disease: Therapeutic implications. Expert Rev Neurother. 2008;8(11):1703‐1718. 18986241 10.1586/14737175.8.11.1703 PMC2631573

[25] Howe FA , Opstad KS . 1H MR spectroscopy of brain tumours and masses. NMR Biomed. 2003;16(3):123‐131. 12884355 10.1002/nbm.822

[26] Bartella L , Huang W . Proton (^1^ H) MR spectroscopy of the breast. Radiographics. 2007;27(suppl_1):S241‐S252. 18180230 10.1148/rg.27si075504

[27] Zhang L , Zhao X , Ouyang H , Wang S , Zhou C . Diagnostic value of 3.0T (1)H MRS with choline‐containing compounds ratio (∆CCC) in primary malignant hepatic tumors. Cancer Imaging. 2016;16(1):25. 27549094 10.1186/s40644-016-0082-4 PMC4994245

[28] Helms G , Piringer A . Restoration of motion‐related signal loss and line‐shape deterioration of proton MR spectra using the residual water as intrinsic reference. Magn Reson Med. 2001;46(2):395‐400. 11477645 10.1002/mrm.1203

[29] Shetty AN , Gabr RE , Rendon DA , Cassady CI , Mehollin‐Ray AR , Lee W . Improving spectral quality in fetal brain magnetic resonance spectroscopy using constructive averaging. Prenat Diagn. 2015;35(13):1294‐1300. 26348874 10.1002/pd.4689

[30] Bolan PJ , Henry P‐G , Baker EH , Meisamy S , Garwood M . Measurement and correction of respiration‐inducedB0 variations in breast1H MRS at 4 Tesla. Magn Reson Med. 2004;52(6):1239‐1245. 15562472 10.1002/mrm.20277

[31] Gabr RE , Sathyanarayana S , Schär M , Weiss RG , Bottomley PA . On restoring motion‐induced signal loss in single‐voxel magnetic resonance spectra. Magn Reson Med. 2006;56(4):754‐760. 16964612 10.1002/mrm.21015 PMC1993303

[32] Star‐Lack JM , Adalsteinsson E , Gold GE , Ikeda DM , Spielman DM . Motion correction and lipid suppression for 1H magnetic resonance spectroscopy. Magn Reson Med. 2000;43(3):325‐330. 10725872 10.1002/(sici)1522-2594(200003)43:3<325::aid-mrm1>3.0.co;2-8

[33] Near J , Edden R , Evans CJ , El Paquin R , Harris A , Jezzard P . Frequency and phase drift correction of magnetic resonance spectroscopy data by spectral registration in the time domain. Magn Reson Med. 73(1):44‐50. 24436292 10.1002/mrm.25094 PMC5851009

[34] Ernst T , Li J . A novel phase and frequency navigator for proton magnetic resonance spectroscopy using water‐suppression cycling. Magn Reson Med. 2011;65(1):13‐17. 20872862 10.1002/mrm.22582 PMC3005004

[35] Dreher W , Leibfritz D . New method for the simultaneous detection of metabolites and water in localized in vivo1H nuclear magnetic resonance spectroscopy. Magn Reson Med. 2005;54(1):190‐195. 15968666 10.1002/mrm.20549

[36] Baumgartner H , Hung J , Bermejo J , et al. Recommendations on the echocardiographic assessment of aortic valve stenosis: A focused update from the European Association of Cardiovascular Imaging and the American Society of Echocardiography. Eur Heart J Cardiovasc Imaging. 2017;18(3):254‐275. 28363204 10.1093/ehjci/jew335

[37] Iung B , Baron G , Butchart EG , et al. A prospective survey of patients with valvular heart disease in Europe: The Euro Heart Survey on valvular heart disease. Eur Heart J. 2003;24(13):1231‐1243. 12831818 10.1016/s0195-668x(03)00201-x

[38] Dahl JS , Eleid MF , Michelena HI , et al. Effect of left ventricular ejection fraction on postoperative outcome in patients with severe aortic stenosis undergoing aortic valve replacement. Circ Cardiovasc Imaging. 2015;8(4). 10.1161/CIRCIMAGING.114.002917 25852129

[39] Ito S , Miranda WR , Nkomo VT , et al. Reduced left ventricular ejection fraction in patients with aortic stenosis. J Am Coll Cardiol. 2018;71(12):1313‐1321. 29566814 10.1016/j.jacc.2018.01.045

[40] Bohbot Y , de Meester de Ravenstein C , Chadha G , et al. Relationship between left ventricular ejection fraction and mortality in asymptomatic and minimally symptomatic patients with severe aortic stenosis. JACC Cardiovasc Imaging. 2019;12(1):38‐48. 30448114 10.1016/j.jcmg.2018.07.029

[41] Peterzan MA , Lygate CA , Neubauer S , Rider OJ . Metabolic remodeling in hypertrophied and failing myocardium: A review. Am J Physiol Heart Circ Physiol. 2017;313(3):H597‐H616. 28646030 10.1152/ajpheart.00731.2016

[42] Peterzan MA , Lewis AJM , Neubauer S , Rider OJ . Non‐invasive investigation of myocardial energetics in cardiac disease using 31P magnetic resonance spectroscopy. Cardiovasc Diagn Ther. 2020;10(3):625‐635. 32695642 10.21037/cdt-20-275 PMC7369290

[43] Peterzan MA , Clarke WT , Lygate CA , et al. Cardiac energetics in patients with aortic stenosis and preserved versus reduced ejection fraction. Circulation. 2020;141(24):1971‐1985. 32438845 10.1161/CIRCULATIONAHA.119.043450 PMC7294745

[44] Bache RJ , Zhang J , Path G , et al. High‐energy phosphate responses to tachycardia and inotropic stimulation in left ventricular hypertrophy. Am J Physiol. 1994;266(5 Pt 2):H1959‐H1970. 8203595 10.1152/ajpheart.1994.266.5.H1959

[45] Lygate CA , Fischer A , Sebag‐Montefiore L , Wallis J , ten Hove M , Neubauer S . The creatine kinase energy transport system in the failing mouse heart. J Mol Cell Cardiol. 2007;42(6):1129‐1136. 17481652 10.1016/j.yjmcc.2007.03.899

[46] Ye Y , Wang C , Zhang J , et al. Myocardial creatine kinase kinetics and isoform expression in hearts with severe LV hypertrophy. Am J Physiol Heart Circ Physiol. 2001;281(1):H376‐H386. 11406506 10.1152/ajpheart.2001.281.1.H376

[47] Tian R , Nascimben L , Ingwall JS , Lorell BH . Failure to maintain a low ADP concentration impairs diastolic function in hypertrophied rat hearts. Circulation. 1997;96(4):1313‐1319. 9286964 10.1161/01.cir.96.4.1313

[48] Ogg RJ , Kingsley PB , Taylor JS . WET, a T1‐ and B1‐insensitive water‐suppression method for in vivo localized 1H NMR spectroscopy. J Magn Reson Ser B. 1994;104(1):1‐10. 8025810 10.1006/jmrb.1994.1048

[49] Thomson LEJ , Kim RJ , Judd RM . Magnetic resonance imaging for the assessment of myocardial viability. J Magn Reson Imaging. 2004;19(6):771‐788. 15170783 10.1002/jmri.20075

[50] Nakae I , Mitsunami K , Matsuo S , et al. Assessment of myocardial creatine concentration in dysfunctional human heart by proton magnetic resonance spectroscopy. Magn Reson Med Sci. 2004;3(1):19‐25. 16093616 10.2463/mrms.3.19

[51] Nakae I , Mitsunami K , Omura T , et al. Proton magnetic resonance spectroscopy can detect creatine depletion associated with the progression of heart failure in cardiomyopathy. J Am Coll Cardiol. 2003;42(9):1587‐1593. 14607443 10.1016/j.jacc.2003.05.005

[52] Weiss K , Martini N , Boesiger P , Kozerke S . Cardiac proton spectroscopy using large coil arrays. NMR Biomed. 2013;26(3):276‐284. 22933454 10.1002/nbm.2845

[53] Liu C‐Y , Redheuil A , Ouwerkerk R , Lima JAC , Bluemke DA . Myocardial fat quantification in humans: Evaluation by two‐point water‐fat imaging and localized proton spectroscopy. Magn Reson Med. 2010;63(4):892‐901. 20373390 10.1002/mrm.22289 PMC3039693

[54] Kankaanpää M , Lehto H‐R , Pärkkä JP , et al. Myocardial triglyceride content and epicardial fat mass in human obesity: relationship to left ventricular function and serum free fatty acid levels. J Clin Endocrinol Metab. 2006;91(11):4689‐4695. 16926257 10.1210/jc.2006-0584

[55] Petritsch B , Köstler H , Weng AM , et al. Myocardial lipid content in Fabry disease: a combined 1H‐MR spectroscopy and MR imaging study at 3 Tesla. BMC Cardiovasc Disord. 2016;16(1):205. 27793097 10.1186/s12872-016-0382-4 PMC5084400

[56] Schär M , Kozerke S , Boesiger P . Navigator gating and volume tracking for double‐triggered cardiac proton spectroscopy at 3 Tesla. Magn Reson Med. 2004;51(6):1091‐1095. 15170826 10.1002/mrm.20123

[57] Rial B . *Development of Proton Magnetic Resonance Spectroscopy in Human Heart at 3 Tesla* [PhD thesis]. 2010. https://ora.ox.ac.uk/objects/uuid:48e60f2d‐ec5c‐4b20‐999a‐b726f8baa436

[58] Rodgers CT , Robson MD . Coil combination for receive array spectroscopy: Are data‐driven methods superior to methods using computed field maps? Magn Reson Med. 2016;75(2):473‐487. 25820303 10.1002/mrm.25618 PMC4744755

[59] Purvis LAB , Clarke WT , Biasiolli L , Valkovič L , Robson MD , Rodgers CT . OXSA: An open‐source magnetic resonance spectroscopy analysis toolbox in MATLAB. PLoS One. 2017;12(9):e0185356. 28938003 10.1371/journal.pone.0185356 PMC5609763

[60] Pijnappel WW , van den Boogaart A , de Beer R , van Ormondt D . SVD‐based quantification of magnetic resonance signals. J Magn Reson. 1992;97(1):122‐134.

[61] Vanhamme F , Van Huffel S , de Beer R . Fast removal of residual water in proton spectra. J Magn Reson. 1998;132(2):197‐203. 9632545 10.1006/jmre.1998.1425

[62] Ernst RR , Bodenhausen G , Wokaun A . Principles of Nuclear Magnetic Resonance in One and Two Dimensions. Oxford: Clarendon Press; 1987.

[63] Cavassila S , Deval S , Huegen C , van Ormondt D , Graveron‐Demilly D . Cramér‐Rao bounds: an evaluation tool for quantitation. NMR Biomed. 2001;14(4):278‐283. 11410946 10.1002/nbm.701

[64] Wang L , Salibi N , Wu Y , Schweitzer ME , Regatte RR . Relaxation times of skeletal muscle metabolites at 7T. J Magn Reson Imaging. 2009;29:1457‐1464. 19472422 10.1002/jmri.21787 PMC2941971

[65] Wang X , Salibi N , Fayad LM , Barker PB . Proton magnetic resonance spectroscopy of skeletal muscle: a comparison of two quantitation techniques. J Magn Reson. 2014;243:81‐84. 24792959 10.1016/j.jmr.2014.03.010 PMC4050659

[66] Reinstein DZ , Archer TJ , Silverman RH , Coleman DJ . Accuracy, repeatability, and reproducibility of Artemis very high‐frequency digital ultrasound arc‐scan lateral dimension measurements. J Cataract Refract Surg. 2006;32(11):1799‐1802. 17081860 10.1016/j.jcrs.2006.07.017 PMC2600896

[67] Bartlett JW , Frost C . Reliability, repeatability and reproducibility: analysis of measurement errors in continuous variables. Ultrasound Obstet Gynecol. 2008;31(4):466‐475. 18306169 10.1002/uog.5256

[68] Faul F , Erdfelder E , Lang AG , Buchner A . G *Power 3: A flexible statistical power analysis program for the social, behavioral, and biomedical sciences. Behav Res Methods. 2007;39:175‐191. 17695343 10.3758/bf03193146

[69] Erdfelder E , FAul F , Buchner A , Lang AG . Statistical power analyses using G*Power 3.1: Tests for correlation and regression analyses. Behav Res Methods. 2009;41(4):1149‐1160. 19897823 10.3758/BRM.41.4.1149

[70] Szczepaniak LS , Dobbins RL , Metzger GJ , et al. Myocardial triglycerides and systolic function in humans: In vivo evaluation by localized proton spectroscopy and cardiac imaging. Magn Reson Med. 2003;49(3):417‐423. 12594743 10.1002/mrm.10372

[71] Felblinger J , Jung B , Slotboom J , Boesch C , Kreis R . Methods and reproducibility of cardiac/respiratory double‐triggered1H‐MR spectroscopy of the human heart. Magn Reson Med. 1999;42(5):903‐910. 10542349 10.1002/(sici)1522-2594(199911)42:5<903::aid-mrm10>3.0.co;2-n

[72] Nakae I , Mitsunami K , Matsuo S , et al. Myocardial creatine concentration in various nonischemic heart diseases assessed by 1H magnetic resonance spectroscopy. Circ J. 2005;69(6):711‐716. 15914951 10.1253/circj.69.711

[73] Mahmod M , Bull S , Suttie JJ , et al. Myocardial steatosis and left ventricular contractile dysfunction in patients with severe aortic stenosis. Circ Cardiovasc Imaging. 2013;6(5):808‐816. 23833283 10.1161/CIRCIMAGING.113.000559

[74] Marfella R , Di Filippo C , Portoghese M , et al. Myocardial lipid accumulation in patients with pressure‐overloaded heart and metabolic syndrome. J Lipid Res. 2009;50(11):2314‐2323. 19470430 10.1194/jlr.P900032-JLR200 PMC2759838

[75] Witteles RM , Fowler MB . Insulin‐resistant cardiomyopathy: clinical evidence, mechanisms, and treatment options. J Am Coll Cardiol. 2008;51(2):93‐102. 18191731 10.1016/j.jacc.2007.10.021

[76] Bottomley PA , Weiss RG . Non‐invasive magnetic‐resonance detection of creatine depletion in non‐viable infarcted myocardium. Lancet. 1998;351(9104):714‐718. 9504516 10.1016/S0140-6736(97)06402-7

[77] Organ CL , Otsuka H , Bhushan S , et al. Choline diet and its gut microbe‐derived metabolite, trimethylamine N‐oxide, exacerbate pressure overload‐induced heart failure. Circ Heart Fail. 2016;9(1):e002314. 26699388 10.1161/CIRCHEARTFAILURE.115.002314 PMC4943035

[78] Wang Z , Klipfell E , Bennett BJ , et al. Gut flora metabolism of phosphatidylcholine promotes cardiovascular disease. Nature. 2011;472(7341):57‐63. 21475195 10.1038/nature09922 PMC3086762

[79] Meyer KA , Shea JW . Dietary choline and betaine and risk of cvd: A systematic review and meta‐analysis of prospective studies. Nutrients. 2017;9(7):711. 10.3390/nu9070711 PMC5537826 28686188

[80] Bidulescu A , Chambless LE , Siega‐Riz AM , Zeisel SH , Heiss G . Usual choline and betaine dietary intake and incident coronary heart disease: The Atherosclerosis Risk in Communities (ARIC) Study. BMC Cardiovasc Disord. 2007;7. 10.1186/1471-2261-7-20 PMC1934379 17629908

[81] Rajaie S , Esmaillzadeh A . Dietary choline and betaine intakes and risk of cardiovascular diseases: review of epidemiological evidence. ARYA Atheroscler. 2011;7(2):78‐86. 22577451 PMC3347848

[82] Zhu W , Wang Z , Tang WHW , Hazen SL . Gut microbe‐generated trimethylamine N‐oxide from dietary choline is prothrombotic in subjects. Circulation. 2017;135(17):1671‐1673. 28438808 10.1161/CIRCULATIONAHA.116.025338 PMC5460631

[83] Millard HR , Musani SK , Dibaba DT , et al. Dietary choline and betaine; associations with subclinical markers of cardiovascular disease risk and incidence of CVD, coronary heart disease and stroke: the Jackson Heart Study. Eur J Nutr. 2018;57(1):51‐60. 27550622 10.1007/s00394-016-1296-8 PMC5931705

[84] Hang P , Zhao J , Su Z , et al. Choline inhibits ischemia‐reperfusion‐induced cardiomyocyte autophagy in rat myocardium by activating Akt/mTOR signaling. Cell Physiol Biochem. 2018;45(5):2136‐2144. 29533930 10.1159/000488049

[85] Liu L , Lu Y , Bi X , et al. Choline ameliorates cardiovascular damage by improving vagal activity and inhibiting the inflammatory response in spontaneously hypertensive rats. Sci Rep. 2017;7(1):42553. 28225018 10.1038/srep42553 PMC5320519

[86] Danne O , Möckel M . Choline in acute coronary syndrome: an emerging biomarker with implications for the integrated assessment of plaque vulnerability. Expert Rev Mol Diagn. 2010;10(2):159‐171. 20214535 10.1586/erm.10.2

[87] Apple FS , Wu AHA , Mair J , et al. Future biomarkers for detection of ischemia and risk stratification in acute coronary syndrome. Clin Chem. 2005;51(5):810‐824. 15774573 10.1373/clinchem.2004.046292

[88] Roy A , Guatimosim S , Prado VF , Gros R , Prado MAM . Cholinergic activity as a new target in diseases of the heart. Mol Med. 2015;20(1):527‐537. 25222914 10.2119/molmed.2014.00125 PMC4365064

[89] Saw EL , Kakinuma Y , Fronius M , Katare R . The non‐neuronal cholinergic system in the heart: A comprehensive review. J Mol Cell Cardiol. 2018;125:129‐139. 30343172 10.1016/j.yjmcc.2018.10.013

[90] Stanisz GJ , Odrobina EE , Pun J , et al. T1, T2 relaxation and magnetization transfer in tissue at 3T. Magn Reson Med. 2005;54(3):507‐512. 16086319 10.1002/mrm.20605

