# Supplementary material for: Water‐suppression cycling 3‐T cardiac 1H‐MRS detects altered creatine and choline in patients with aortic or mitral stenosis
Source: NMR Biomed. 2021 Apr 7;34(7):e4513. doi: 10.1002/nbm.4513 (PMC8243349; doi:10.1002/nbm.4513)
Supplement: Supplementary file 1 — Figure S1: Simulation workflow and results showing that amplitudes of metabolite peaks remain unchanged by the weighting factor. The scatter plot at the bottom shows the mean and standard deviation in percentage difference across all 200 sets for each metabolite and each w factor and a reference set (where only the noise varied). Figure S2: Six compartment phantom in silicone ice cube tray with six different concentrations of creatine (0, 5, 10, 20, 40, and 80 mmol/L). Each compartment is roughly 4.8 × 4.8 × 4.8 cm3. Figure S3: Cardiac spectra of a healthy volunteer obtained by PRESS‐WET (green) and STEAM‐WET (blue) showing the various metabolite peaks: CH2 of total creatine (Cr CH2), choline (Cho), CH3 of total creatine (Cr CH3) and lipid peaks. On average across six healthy volunteers, PRESS‐WET had 23% smaller SNR and 88% larger CRLB value for the Cr CH3 peak compared to STEAM‐WET. Figure S4: SNR (A), CRLB (B) and linewidths (C) for the first (red), last (blue) and 3 random sets (green) of 12 breathholds obtained from the STEAM‐WET (150 meas.) acquisition in healthy volunteers. The random set corresponding to the data set used in the paper is labelled as ‘Random 1’ and plotted in a darker shade of green. Each volunteer is represented by a different symbol and data points more than 1.5 interquartile ranges above the upper quartile or below the lower quartile are treated as outliers (coloured black). The black cross and corresponding whiskers show the mean and standard deviation for each data set. No significant difference was found between any of the parameters in the various sets of 12 breathholds. Table S1: Median and IQR of (A) SNR, (B) CRLB and (C) linewidths of fitted peaks in the STEAM‐WET vs PRESS‐WET pre‐study pilot experiment involving 6 healthy volunteers. Table S2: Table summarising patient demographics. Values are given as number(%), mean (SD) or median (quartile 1 – quartile 3). Table S3: SNR and CRLB of each metabolite peak in the phantom. The measur [file NBM-34-e4513-s001.docx]

## Supplementary information


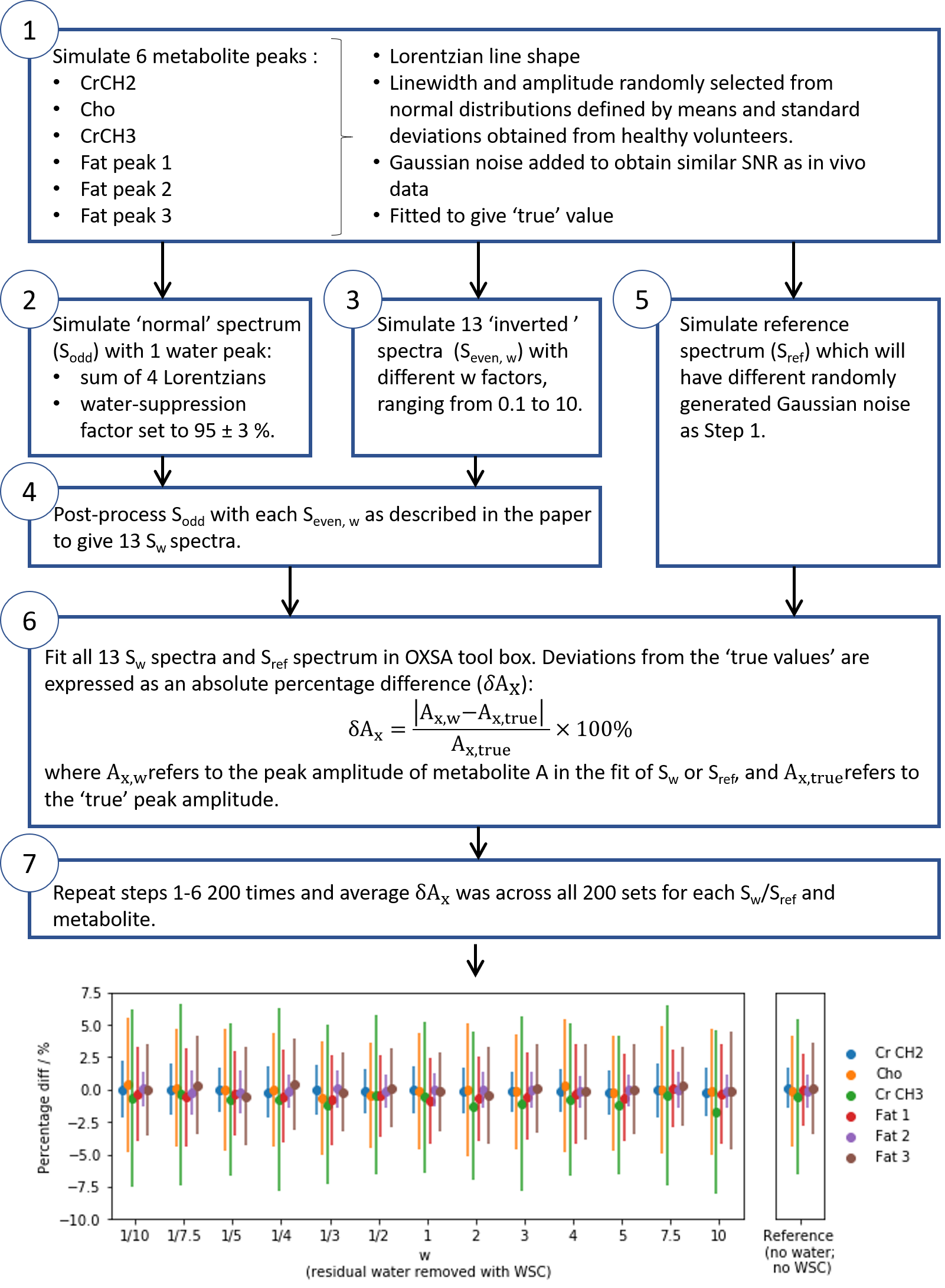


SI Figure 1: Simulation workflow and results showing that amplitudes of metabolite peaks remain unchanged by the weighting factor. The scatter plot at the bottom shows the mean and standard deviation in percentage difference across all 200 sets for each metabolite and each w factor and a reference set (where only the noise varied).


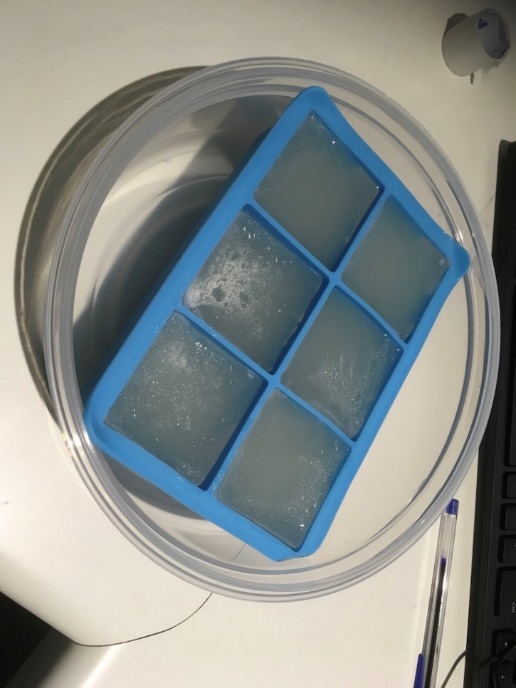


SI Figure 2: Six compartment phantom in silicone ice cube tray with six different concentrations of creatine (0, 5, 10, 20, 40, and 80 mmol/L). Each compartment is roughly 4.8 × 4.8 × 4.8 cm^3^.


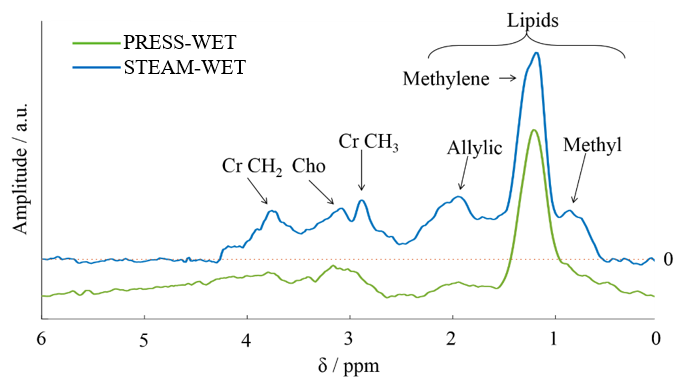


SI Figure 3: Cardiac spectra of a healthy volunteer obtained by PRESS-WET (green) and STEAM-WET (blue) showing the various metabolite peaks: CH_2_ of total creatine (Cr CH_2_), choline (Cho), CH_3_ of total creatine (Cr CH_3_) and lipid peaks. On average across six healthy volunteers, PRESS-WET had 23% smaller SNR and 88% larger CRLB value for the Cr CH_3_ peak compared to STEAM-WET.


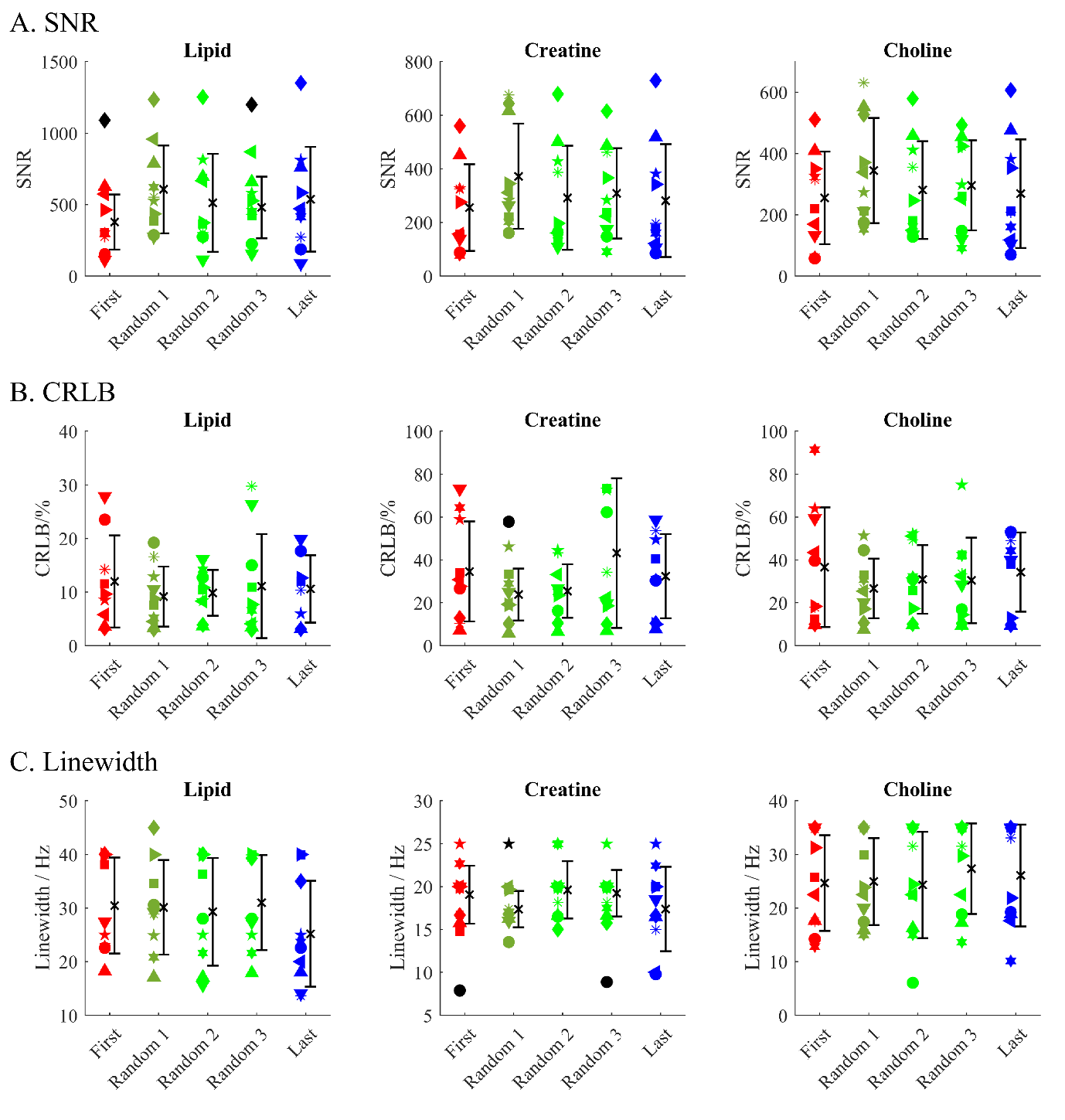


SI Figure 4: SNR (A), CRLB (B) and linewidths (C) for the first (red), last (blue) and 3 random sets (green) of 12 breathholds obtained from the STEAM-WET (150 meas.) acquisition in healthy volunteers. The random set corresponding to the data set used in the paper is labelled as ‘Random 1’ and plotted in a darker shade of green. Each volunteer is represented by a different symbol and data points more than 1.5 interquartile ranges above the upper quartile or below the lower quartile are treated as outliers (coloured black). The black cross and corresponding whiskers show the mean and standard deviation for each data set. No significant difference was found between any of the parameters in the various sets of 12 breathholds.

SI Table 1: Median and IQR of (A) SNR, (B) CRLB and (C) linewidths of fitted peaks in the STEAM-WET vs PRESS-WET pre-study pilot experiment involving 6 healthy volunteers.

| 1. Median (IQR) of SNR | | | | | |
| --- | --- | --- | --- | --- | --- |
| Group | Sequence | Meas. | Lipid | Creatine | Choline |
| Healthy  n = 6 | STEAM-WET | 150 | 953 (611 – 1574) | 511 (275 – 625) | 520 (283 – 584) |
|  | PRESS-WET | 150 | 744 (180 – 1240) | 356 (157 – 390) | 297 (223 – 386) |
| B) Median (IQR) of CRLB / % | | | | | |
| Group | Sequence | Meas. | Lipid | Creatine | Choline |
| Healthy  n = 6 | STEAM-WET | 150 | 10.5 (4.1 – 20.9) | 14.7 (11.4 – 25.9) | 18.2 (9.4 – 36.9) |
|  | PRESS-WET | 150 | 55.6 (34.0 – 79.4) | 18.8 (13.5 – 57.2) | 19.6 (17.7 – 88.6) |
| C) Median (IQR) of linewidth / Hz | | | | | |
| Group | Sequence | Meas. | Lipid | Creatine | Choline |
| Healthy  n = 6 | STEAM-WET | 150 | 28.7 (23.8 – 38.1) | 14.4 (11.9 – 19.0) | 19.2 (16.2 – 19.5) |
|  | PRESS-WET | 150 | 47.1 (37.3 – 117.4) | 41.8 (21.2 – 47.3) | 41.2 (26.3 – 53.1) |

SI Table 2: Table summarising patient demographics. Values are given as number(%), mean (SD) or median (quartile 1 – quartile 3).

| n | 13 |
| --- | --- |
| Male | 10 (77%) |
| Age | 76 (7) |
| NYHA class | 1.9 (0.4) |
| Body mass index (kg/m^2^) | 27 (4) |
| Body surface area (m^2^) | 1.95 (0.22) |
| Heart rate (/min) | 66 (61 – 76) |
| Systolic blood pressure (mmHg) | 140 (27) |
| Diastolic blood pressure (mmHg) | 73 (9) |
| Mean arterial pressure (mmHg) | 96 (12) |
| LV end-diastolic volume index (ml/m^2^) | 79 (15) |
| LV ejection fraction (%) | 60 (10) |
| LV mass index (g/m^2^) | 83 (27) |
| Biopsy creatine (nmol/mg protein) | 53 (18) |
| Time between MRI /MRS scan and biopsy (days) | 7 (1 – 12) |

SI Table 3: SNR and CRLB of each metabolite peak in the phantom. The measurements were obtained from the compartment containing 80 mmol/L of Cr. All acquisition parameters were as described in the Methods section of the paper, except a TE of 40 ms was used for all protocols to ensure a fairer comparison of SNR.

| Mean SNR (CRLB / %) | | | | | |
| --- | --- | --- | --- | --- | --- |
| Sequence | Measurements | Creatine CH_2_  (3.7 ppm) | Creatine CH_3_  (3.0 ppm) | Lipid peak 1  (1.28 ppm) | Lipid peak 2  (0.84 ppm) |
| PRESS-WET | 30 | 6068 (1.3 %) | 10937 (0.7 %) | 9986 (0.9 %) | 4071 (19.8 %) |
| STEAM-WET | 30 | 3267 (1.2 %) | 5999 (0.6 %) | 6100 (0.6 %) | 1626 (3.8 %) |
| PRESS-WSC | 30 | 5463 (1.2 %) | 10171 (0.6 %) | 9397 (0.8 %) | 3572 (13.3 %) |
| STEAM-WSC | 30 | 2674 (1.4 %) | 5192 (0.7 %) | 5607 (0.6 %) | 1474 (5.2 %) |

SI Table 4: Medians along with interquartile ranges (IQR) of SNR, CRLB and linewidths over all n subjects are shown in (A), (B) and (C) respectively.

| 1. Median (IQR) of SNR | | | | | |
| --- | --- | --- | --- | --- | --- |
| Group | Sequence | Meas. | Lipid | Creatine | Choline |
| Healthy  n = 10 | STEAM-WET-150 | 150 | 801 (602 – 1093) | 410 (293 – 600) | 423 (294 – 533) |
|  | STEAM-WET-60 | 60 | 539 (398 – 747) | 298 (232 – 548) | 306 (212 – 488) |
|  | STEAM-WSC | 60 | 389 (322 – 858) | 214 (155 – 456) | 222 (151 – 439) |
|  | PRESS-WSC | 60 | 812 (580 – 1025) | 449 (328 – 556) | 426 (329 – 514) |
| Patients  n = 8 | STEAM-WET-150 | 150 | 1878 (1088 – 2301) | 461 (325 – 508) | 440 (357 – 524) |
|  | STEAM-WET-60 | 60 | 1054 (644 – 1536) | 296 (222 – 373) | 296 (224 – 356) |
|  | PRESS-WSC | 60 | 1078 (945 – 1415) | 268 (247 – 478) | 305 (252 – 463) |
| B) Median (IQR) of CRLB / % | | | | | |
| Group | Sequence | Meas. | Lipid | Creatine | Choline |
| Healthy  n = 10 | STEAM-WET-150 | 150 | 8.4 (4.5 – 16.4) | 18.9 (12.7 – 37.2) | 20.1 (11.9 – 39.7) |
|  | STEAM-WET-60 | 60 | 8.2 (4.7 – 12.3) | 25.8 (19.1 – 32.3) | 26.0 (17.9 – 32.3) |
|  | STEAM-WSC | 60 | 7.1 (3.6 – 12.2) | 18.8 (11.2 – 29.7) | 20.6 (12.4 – 25.8) |
|  | PRESS-WSC | 60 | 6.2 (3.4 – 8.2) | 13.2 (9.0 – 16.8) | 13.7 (7.4 – 17.8) |
| Patients  n = 8 | STEAM-WET-150 | 150 | 31.3 (12.3 – 41.1) | 12.9 (7.2 – 15.9) | 21.5 (9.3 – 39.6) |
|  | STEAM-WET-60 | 60 | 36.3 (26.1 – 51.8) | 17.3 (14.5 – 47.0) | 40.1 (25.2 – 97.7) |
|  | PRESS-WSC | 60 | 27.4 (10.0 – 80.3) | 14.4 (13.2 – 65.6) | 23.1 (15.0 – 73.1) |
| C) Median (IQR) of linewidth / Hz | | | | | |
| Group | Sequence | Meas. | Lipid | Creatine | Choline |
| Healthy  n = 10 | STEAM-WET-150 | 150 | 36.8 (31.4 – 39.4) | 18.9 (15.6 – 21.1) | 20.0 (17.3 – 20.0) |
|  | STEAM-WET-60 | 60 | 29.9 (25.9 – 38.6) | 17.2 (16.4 – 19.7) | 23.2 (18.1 – 33.7) |
|  | STEAM-WSC | 60 | 13.7 (10.1 – 19.0) | 5.3 (4.9 – 7.7) | 13.1 (7.5 – 17.5) |
|  | PRESS-WSC | 60 | 24.5 (20.6 – 28.8) | 11.9 (10.0 – 15.4) | 17.4 (10.0 – 19.4) |
| Patients  n = 8 | STEAM-WET-150 | 150 | 32.9 (29.1 – 40.0) | 12.6 (10.7 – 17.5) | 27.4 (18.1 – 31.1) |
|  | STEAM-WET-60 | 60 | 33.2 (23.3 – 39.3) | 17.3 (9.8 – 20.0) | 30.1 (23.9 – 35.0) |
|  | PRESS-WSC | 60 | 27.7 (16.6 – 34.0) | 15.9 (10.6 – 19.1) | 21.1 (18.7 – 25.4) |
